# Nitrate contamination in drinking water and adverse reproductive and birth outcomes: a systematic review and meta-analysis

**DOI:** 10.1038/s41598-022-27345-x

**Published:** 2023-01-11

**Authors:** Luling Lin, Sophie St Clair, Greg D. Gamble, Caroline A. Crowther, Lesley Dixon, Frank H. Bloomfield, Jane E. Harding

**Affiliations:** 1 Liggins Institute, University of Auckland, Auckland, New Zealand; 2 New Zealand College of Midwives, 376 Manchester Street, Richmond, Christchurch, 8014 New Zealand

**Keywords:** Environmental monitoring, Preterm birth, Epidemiology

## Abstract

Exposure to low levels of nitrate in drinking water may have adverse reproductive effects. We reviewed evidence about the association between nitrate in drinking water and adverse reproductive outcomes published to November 2022. Randomized trials, cohort or case–control studies published in English that reported the relationship between nitrate intake from drinking water and the risk of perinatal outcomes were included. Random-effect models were used to pool data. Three cohort studies showed nitrate in drinking water is associated with an increased risk of preterm birth (odds ratio for 1 mg/L NO_3_-N increased (OR_1_) = 1.01, 95% CI 1.00, 1.01, I^2^ = 23.9%, 5,014,487 participants; comparing the highest versus the lowest nitrate exposure groups pooled OR (OR_p_) = 1.05, 95% CI 1.01, 1.10, I^2^ = 0%, 4,152,348 participants). Case–control studies showed nitrate in drinking water may be associated with the increased risk of neural tube defects OR_1_ = 1.06, 95% CI 1.02, 1.10; 2 studies, 2196 participants; I^2^ = 0%; and OR_p_ = 1.51, 95% CI 1.12, 2.05; 3 studies, 1501 participants; I^2^ = 0%). The evidence for an association between nitrate in drinking water and risk of small for gestational age infants, any birth defects, or any congenital heart defects was inconsistent. Increased nitrate in drinking water may be associated with an increased risk of preterm birth and some specific congenital anomalies. These findings warrant regular review as new evidence becomes available.

## Introduction

Nitrate is a water-soluble ion made up of nitrogen and oxygen with the chemical formula NO_3_^−^. It is a naturally occurring ion that is part of the nitrogen cycle involving the interchange of nitrogen between the atmosphere, land and living organisms^1^. In humans the main intake of nitrates is from food; vegetables constitute at least 85% of nitrate consumption^2^. Drinking water normally contributes only a small percentage of total nitrate intake based on consumption habits. For example, in New Zealand, less than 10% of total nitrate intake is from drinking water, with most of the remainder coming from the diet^3^. However, if the nitrate concentration in drinking water is high, it may contribute a much larger proportion of total nitrate intake.

Nitrogen is very important for plant nutrition and growth, being incorporated by plants into amino acid synthesis, and is therefore commonly used in inorganic fertilizers. However, because nitrate is highly water soluble, it leaches through soils and into groundwater very easily, particularly after heavy rainfall. About 80–90% of the world’s freshwater comes from groundwater^4^, and 50% of the total population relies on groundwater for daily drinking water^5^. The increasing use of artificial fertilizers, the disposal of wastes, particularly from animal farming, and changes in land use have become significant contributors to the progressive increase in nitrate levels in groundwater supplies^1^.

The current World Health Organization (WHO) guideline value for nitrate in drinking water is 50 mg/L as nitrate (NO_3_^−^) or 11.3 mg/L as nitrate-nitrogen (NO_3_-N) (multiply NO_3_^−^ mg/L by 0.2259)^1^. This concentration is approximately equivalent to the current U.S. federal maximum contaminant level (MCL) for nitrate in public drinking water supply of 10 mg/L as NO_3_-N. This limit was established to protect against methemoglobinemia in infants, or blue baby syndrome, the most widely recognized health consequence of high nitrate exposure^6^.

While there is some evidence that nitrate in drinking water is associated with colorectal cancer^7–11^, the potential for adverse reproductive effects of chronic exposure to low levels of nitrate has also been raised recently^12–15^. Animal studies have indicated that nitrate from the mother can cross the placenta, affect the fetus in utero, and increase adverse outcomes, such as abortion, birth defects, gastroschisis, microphthalmia, anophthalmia, and craniofacial hypoplasia^16–20^.

The proposed mechanism for nitrate to cause adverse birth outcomes is via its reduction to nitrite, leading to the transformation of hemoglobin to methemoglobin, which cannot carry oxygen, thus reducing transfer of oxygen to body cells^21,22^. It has been suggested that fetal plasma nitrate levels may be higher than those of the mother because nitrate or nitrite can transfer to the fetus^23^ and fetal hemogolobin is especially vulnerable to oxidation. In addition, antioxidant defence is relatively deficient in newborns and, even when antioxidants are present, they do not fully mitigate oxidative/nitrosative stress or its consequences^24^. Further, since water does not contain antioxidants, nitrate intake from drinking water may be more damaging than that consumed from food which may contain antioxidants. Other proposed mechanisms for the potential effects of nitrate on reproductive health including the creation of N-nitroso compounds and thyroid and endocrine disturbance^8,25^.

Several epidemiological studies in humans have reported an association between prenatal nitrate exposure and adverse reproductive outcomes, including congenital abnormalities, preterm birth, low birth weight and small-for-gestational-age (SGA) infants^13,26–29^. Two previous systematic reviews have assessed the association between maternal nitrate intake and risk of neural tube defects^30^, birth defects, and preterm birth^31^, but the exposures included nitrate intake from food and drugs as well as drinking water.

The purpose of this study was to systematically review the published evidence to determine the association between human exposure to nitrate in drinking water and adverse reproductive and birth outcomes. This study was focused on the international context but uses New Zealand examples.

## Results

### Search results, study characteristics and quality

**Figure 1:**
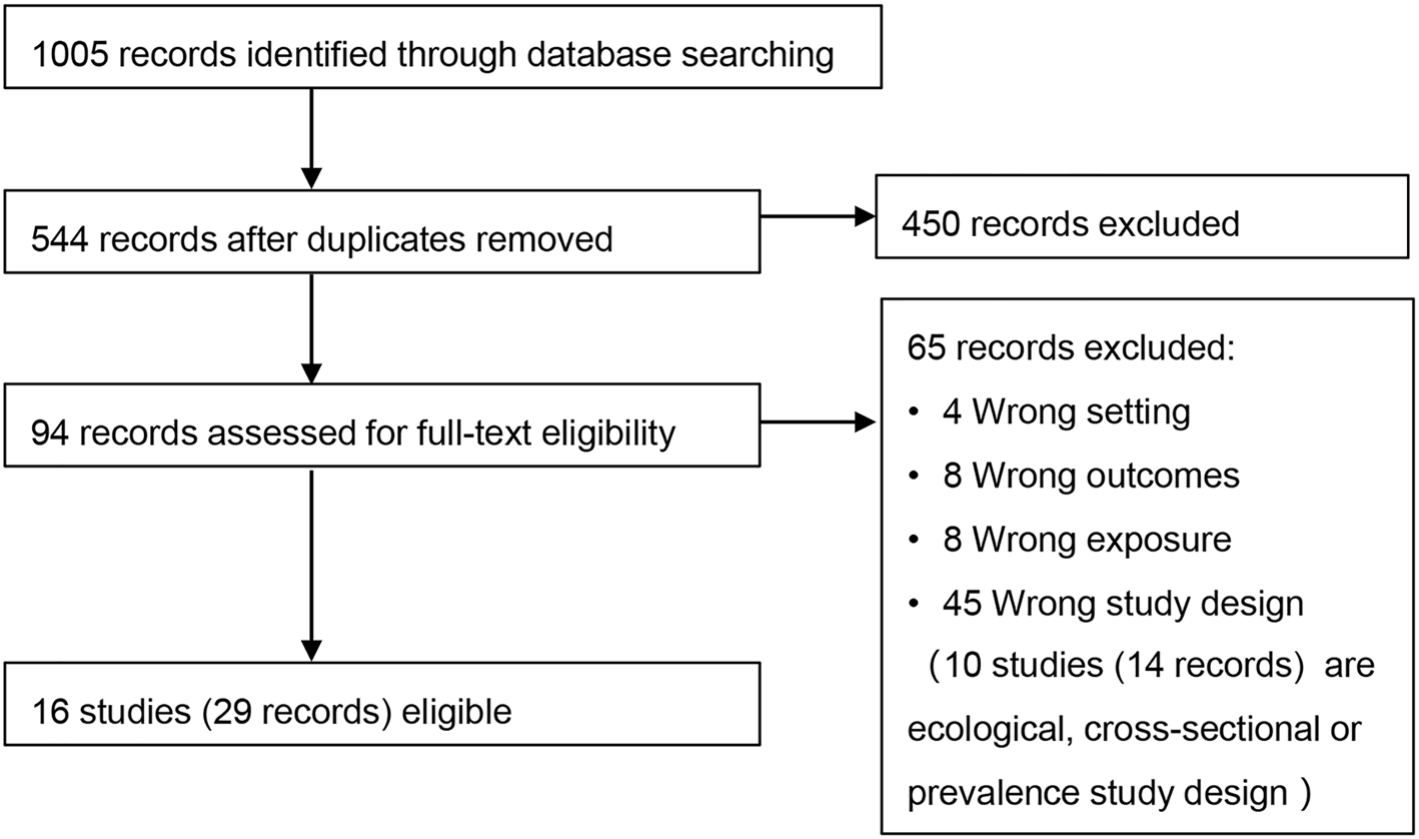
Flow diagram of included studies.

In total, 1005 records were identified from database searching. After removal of the duplicates, we completed title and abstract screening for 544 records and then full-text screening for 94 records, of which 65 did not meet our inclusion criteria. Sixteen studies (29 records) met our inclusion criteria (Fig. 1).

Among the 65 records that did not meet the study design criteria for this review, we identified six ecological, three cross-sectional and one record-based prevalence study exploring the association between nitrate exposure in drinking water and adverse reproductive and birth outcomes. These ten studies were included in the qualitative analysis and are presented as additional evidence.

The 16 studies included in the analysis were published between 1982 and 2022 and included 7,268,991 participants (range from 394 to 4,160,998). Five were cohort studies carried out in Denmark(2), France(1), US(1) and Sweden(1). The other eleven were case–control studies in US(7), Canada(2), Australia(1) and Sweden(1). The study characteristics are described in Table 1.

**Table 1:** Study characteristics.

Study name	Country, Region	Study design	Years of outcome ascertainment	Exposure description	Perinatal outcomes reported
Cedergren 2002^32^	Sweden, Östergötland County	Retrospective cohort study	1982–1996	Maternal addresses linked to water supplies using a geographic information system Exposure measured period: samples represent nitrates in drinking water in either periconceptional or pregnancy period	Any cardiac defect
Ebdrup 2022^33^	Denmark	Cohort study	1996–2002	Nitrate in drinking water estimates were taken from the national drinking water quality monitoring database, Jupiter. Individual-level household exposure estimates were obtained through the geocoded residential history for every person registered in the Danish Civil Registration System, and these estimates were linked with water supply areas Exposure measure period: the date of the last menstrual period (LMP) to the date of pregnancy outcome or end of follow-up, whichever came first	Spontaneous pregnancy losses
Sherris 2021^34^	US, California	Retrospective cohort study	2000–2011	Geocoded residences were linked to water supplies, and public monitoring records of nitrate levels were used. Births were then assigned to exposure categories (low, medium, high) Exposure measured period: duration of the pregnancy	Preterm birth
**Stayner 2022**					
Coffman 2021^13^	Denmark	Prospective cohort study	1991–2011	Nitrate in drinking water estimates were taken from the Danish national geodatabase, Jupiter. The residential addresses of mothers were taken from the Danish Civil Registration System. Exposure was assigned per month of pregnancy and then time-weighted averages used to calculate an overall pregnancy exposure. Data linkage was done using the unique personal identification number assigned to each resident in Denmark Exposure measured period: duration of the pregnancy	Low birth weight, birth weight, body length, head circumference
Coffman 2022^35^			1991–2015	Unique personal identification number assigned to each liveborn resident in Denmark linked to household level of nitrate in drinking water from the Danish national monitoring geodatabase Jupiter and Danish Medical Birth Registry Exposure measured period: averaged nitrate over the pregnancy (accounting for changes in maternal address)	Preterm birth
Stayner 2022^36^			1991–2013	Maternal addresses linked to the national monitoring database, Jupiter, which contains drinking water monitoring data Exposure measured period: averaged nitrate over the pregnancy (accounting for changes in maternal address)	Birth defects
Thomsen 2021^37^			1997–2017	Maternal addresses from Danish Civil Registration System linked to the national monitoring database, Jupiter, which contains drinking water monitoring data Exposure measured period: first 22 weeks of pregnancy	Stillbirth
**Migeot 2013**					
Migeot 2013^38^	France, Deux-Sèvres	Historic cohort study	2005–2009	Measurements of nitrate in community water systems (263 municipalities) were linked to maternal place of residence on the date of birth Exposure measured period: second trimester (taking season into account)	SGA births
Limousi 2014^39^			2005–2010		SGA births
Albouy-Llaty 2016^40^			2005–2010		Preterm birth
Arbuckle 1988^41^	Canada, New Brunswick	Population- based case–control study	1973–1983	Water samples (3 samples of flushed drinking water) from households of the study subjects were collected to estimate the nitrate concentrations Exposure measured period: not specified	Central nervous system malformation
Aschengrau 1989^42^	US, Massachusetts, Boston	Case–control study	1976–1978	Residential addresses at the time of pregnancy matched to routinely collected drinking water data. The information on drinking water source (surface, ground, or mixed) and treatment (chlorination or chloramination) for surface water were considered Exposure measured period: not specified	Spontaneous abortion
Aschengrau 1993^43^	US, Massachusetts, Boston	Case–control study	1977–1980	Residential addresses at the time of pregnancy matched to routinely collected drinking water data Exposure measured period: during the first trimester	Congenital anomaly, stillbirth, neonatal death
Brender 2004^44^	US, Texas-Mexico Border Counties	Case–control study	1995–2000	Water samples collected from the residential address were measured for nitrates Exposure measured period: not specified, samples meant to approximate pregnancy period	Neural tube defects
Brender 2013^28^	US, Iowa and Texas	Population-based case–control study	1997–2005	Maternal addresses linked to public water utility nitrate measurements; nitrate ingestion (NO_3_^−^) estimated from reported water consumption Exposure measured period: 1 month before conception through the end of the third month of pregnancy; or 1 month before conception through 1-month post-conception for neural tube defects	Neural tube defects, limb deficiencies, oral cleft defects, congenital heart defects
Croen 2001^45^	US, California	Population-based case–control study	1989–1991	Maternal addresses linked to water companies by city or Department of Health Services Water Quality Monitoring Database Exposure measured period: periconceptional period	Neural tube defects
Dorsch 1984^46^	Australia, Mount Gambier	Case–control study	1951–1979	Maternal addresses at the time of admission to hospital linked to water source Exposure measured period: samples assumed to represent exposure during pregnancy	Congenital malformation
Ericson 1988^47^	Sweden	Case–control study	1976–1977	Earliest known maternal addresses linked to data from county environmental surveillance offices at the county councils Exposure measured period: assumed to represent exposure during pregnancy	Neural tube defects
Holtby 2014^27^	Canada, Kings County, Nova Scotia	Population-based case–control study	1988–2006	Maternal addresses at birth linked to municipal water supply; the median of all nitrate concentration measurements taken within each municipal water supply was used as the nitrate exposure estimate for all study participants living in each municipality. Nitrate in rural private wells was estimated using geographic information system from the nitrate concentrations of monthly samples taken. The latitude and longitude of the maternal address at the time of delivery was then used to determine a nitrate-exposure estimate for each study participant Exposure measured period: not specified	Congenital malformations as a single group
Liu 2008^48^	US, Connecticut	Case–control study	2002–2004	Maternal addresses from birth certificates linked to public drinking water data from the Connecticut Department of Public Health Exposure measured period: not specified	Birth defect, low birth weight, preterm birth
Waller 2010^49^	US, Washington State	Retrospective case–control study	1987–2006	The distance between maternal residence and the closest stream monitor site with nitrate > 10 mg/L as NO_3_-N were used to estimate the risk Exposure measured period: not specified	Gastroschisis

The cohorts reported by Migeot 2013, Limousi 2014 and Albouy-Llaty 2016 are births in the same place in different periods, but there are overlaps between these three cohorts. The cohorts reported by Coffman 2021, Coffman 2022, Stayner 2022 and Thomsen 2021 are births in the same place in difference periods, but there are overlaps between these four cohorts. SGA = small-for-gestational-age.

According to the Newcastle–Ottawa Scale (NOS) (Table 2), all five cohort studies and four of the eleven case–control studies were considered of high quality; seven of the eleven case–control studies were assessed as moderate study quality.

**Table 2:** Quality of the included studies using the Newcastle–Ottawa scale.

	Selection	Comparability	Outcome	Total
**Cohort study**				
Cedergren 2002	★★★★	★★	★	7
Ebdrup 2022	★★★★	★★	★★★	9
Migeot 2013				
Migeot 2013	★★★★	★★	★★	8
Limousi 2014	★★★★	★★	★★	8
Albouy-Llaty 2016	★★★★	★★	★★	8
Sherris 2021	★★★★	★★	★★	8
Stayner 2022				
Stayner 2022	★★★	★★	★★★	8
Coffman 2022	★★★	★★	★★★	8
Coffman 2021	★★★	★★	★★★	8
Thomsen 2021	★★★	★★	★★★	8
**Case–control study**				
Arbuckle 1988	★★	★	★★	5
Aschengrau 1989	★★	★★	★★	6
Aschengrau 1993	★★	★★	★	5
Brender 2004	★★★★	★	★	6
Brender 2013	★★★	★★	★★	7
Croen 2001	★	★	★★★	5
Dorsch 1984	★★	★★	★★★	7
Ericson 1988	★★	★★	★★	6
Holtby 2014	★★★	★★	★★	7
Liu 2008	★★	★	★★	5
Waller 2010	★★★★	★★	★★	7

Scale is from 0–9, where values ≥ 7 are compatible with good study quality.

### Primary outcome

Four studies reported the odds ratios (ORs) for preterm birth, one study reported the ORs for SGA and two studies reported the ORs for low birth weight, but it was not possible to pool data about the composite of any of these outcomes.

### Secondary outcomes

#### Preterm birth

**Figure 2:**
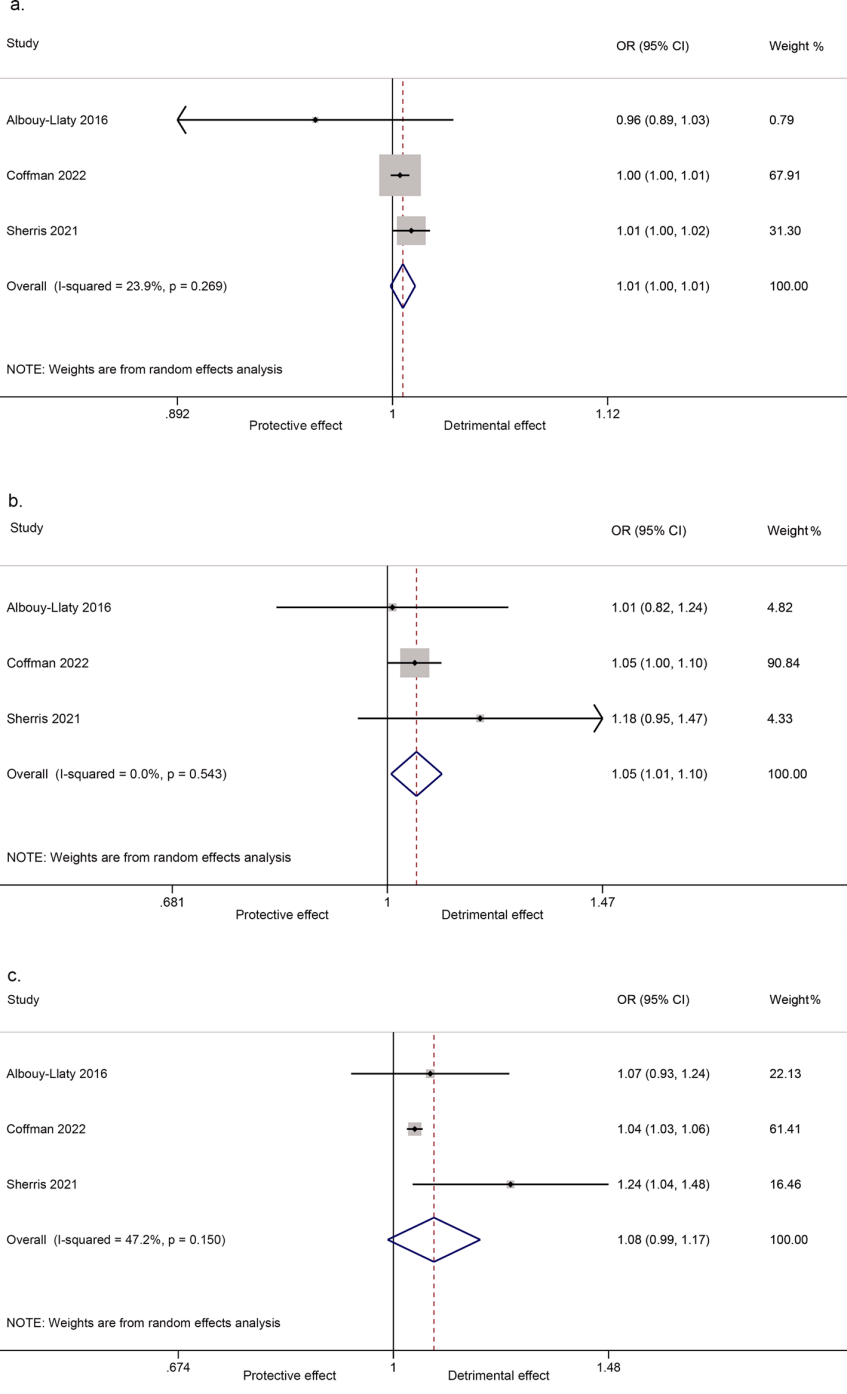
Forest plot for the association between nitrate in drinking water and preterm birth. (**a**) Linear relationship; (**b**) Highest versus lowest exposure; (**c**) All combined higher versus the lowest exposure.

When analyzed as a linear relationship, there was an association between nitrate in drinking water and preterm birth (3 cohort studies, 5,014,487 participants; OR for 1 mg/L (OR_1_) = 1.01, 95% CI 1.00, 1.01; I^2^ = 23.9%, Fig. 2a). There also was an association when comparing the highest nitrate exposure to the lowest exposure (3 cohort studies, 4,152,348 participants; pooled OR (OR_p_) = 1.05, 95% CI 1.01, 1.10, I^2^ = 0%, Fig. 2b). When comparing the combined higher nitrate exposures to the lowest exposure did not show a significant association (3 cohort studies, 5,023,271 participants; OR_p_ = 1.08, 95% CI 0.99, 1.17; I^2^ = 47.2%, Fig. 2c). However, one case–control study^48^ reported that there was no association between nitrate in drinking water and preterm birth.

#### Any birth defects

**Figure 3:**
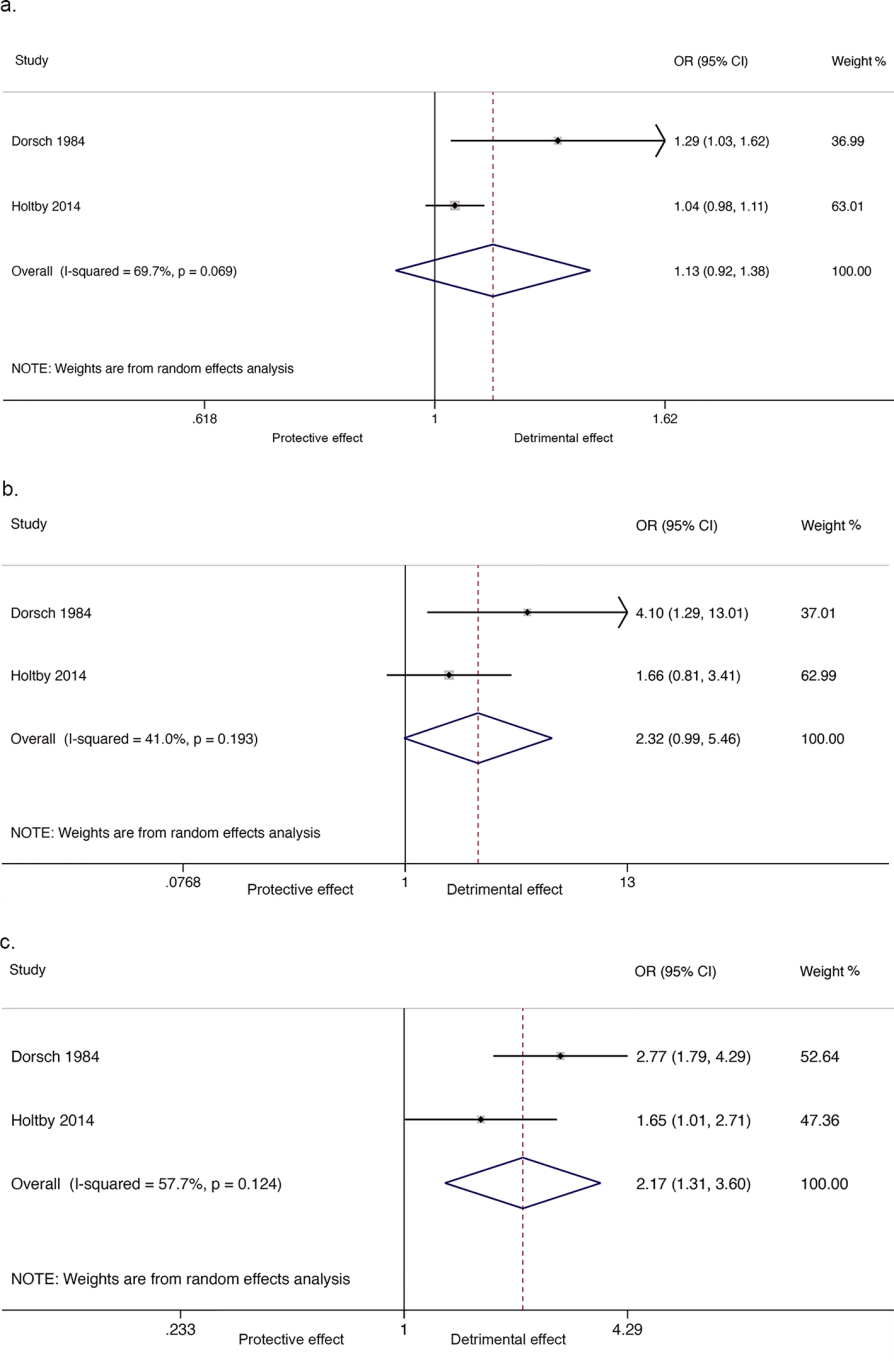
Forest plot for the association between nitrate in drinking water and any birth defects. (**a**) Linear relationship; (**b**) Highest versus the lowest exposure; (**c**) All combined higher versus the lowest exposure.

One cohort study^36^ reported a weak inverse association between nitrate in drinking water and any birth defect when treating nitrate as a continuous variable (1 cohort study, 1,018,914 participants; OR_1_ = 0.98, 95% CI 0.97, 1.00). When analyzed as a linear relationship, findings from two case–control studies^27,46^ did not show an association between nitrate in drinking water and any birth defect (2 case–control studies, 2676 participants; OR_1_ = 1.13, 95% CI 0.92, 1.38; I^2^ = 69.7%; Fig. 3a); however, significant heterogeneity was present. Comparing the highest to the lowest nitrate exposure groups did not show a significant association between nitrate in drinking water and any birth defects (2 case–control studies, 1150 participants, OR_p_ = 2.32, 95% CI 0.99, 5.42; I^2^ = 41.0%; Fig. 3b), but comparing all combined higher exposures to the lowest exposure group showed evidence of an association between the risk of any birth defect and nitrate in drinking water (2 case–control studies, 2676 participants; OR_p_ = 2.17, 95% CI 1.31, 3.60; I^2^ = 57.7%, Fig. 3c). Aschengrau^43^ reported there was no evidence of an association between detectable nitrate concentration in drinking water and any birth defects.

#### Neural tube defects

**Figure 4:**
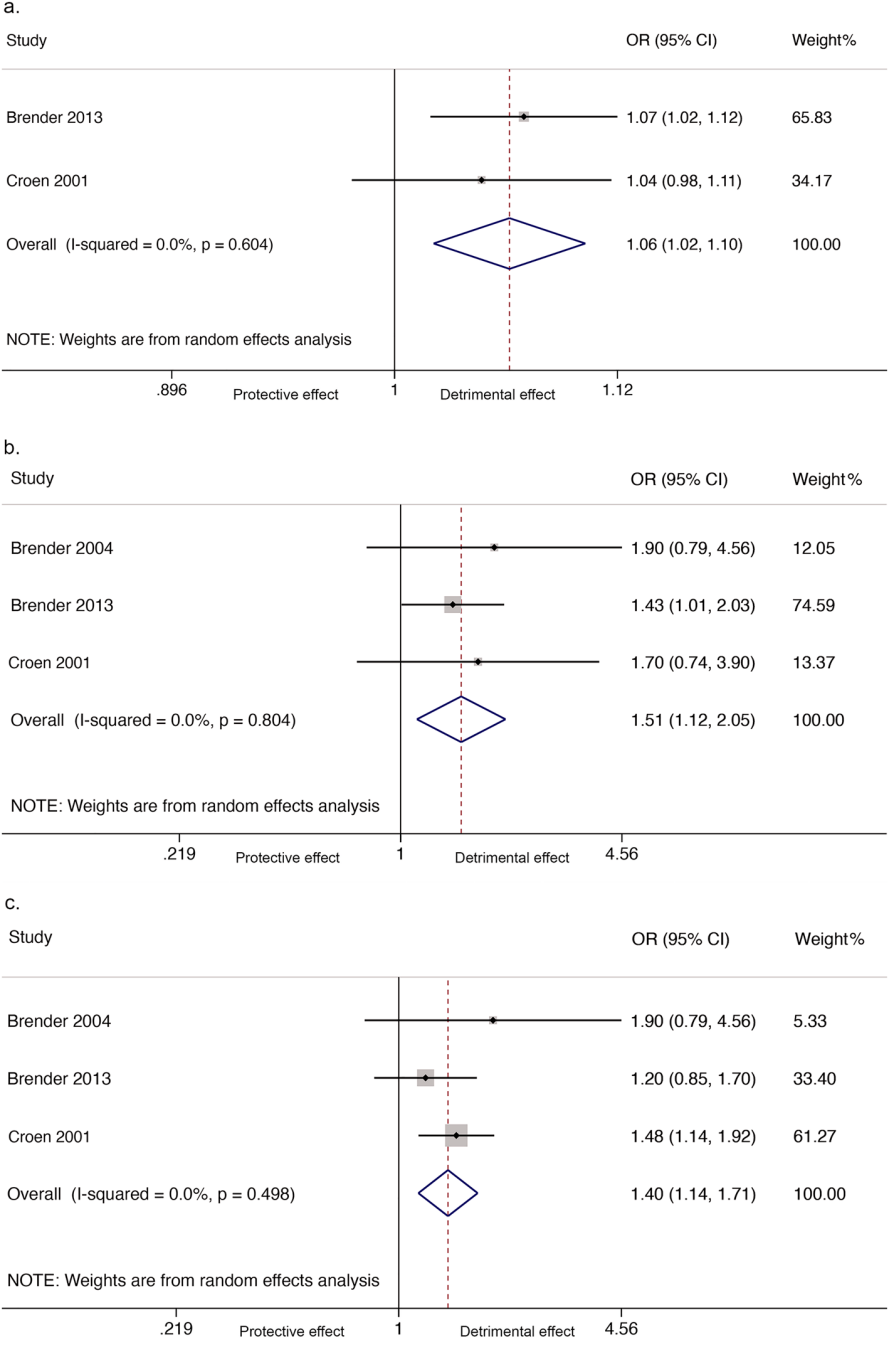
Forest plot for the association between nitrate in drinking water and neural tube defects. (**a**) Linear relationship; (**b**) Highest versus the lowest exposure; (**c**) All combined higher versus the lowest exposure**.**

One cohort study^36^ reported there was no evidence of an association between nitrate in drinking water and any neural tube defects. However, when analyzed as a linear relationship, findings from case–control studies showed a significant relationship between nitrate in drinking water and neural tube defects (2 studies, 2,196 participants; OR_1_ = 1.06, 95% CI 1.02, 1.10; I^2^ = 0%; Fig. 4a). There also was an association between nitrate in drinking water and neural tube defects when comparing the highest versus the lowest nitrate exposure groups (3 studies, 1,501 participants; OR_p_ = 1.51, 95% CI 1.12, 2.05; I^2^ = 0%; Fig. 4b) and when comparing all combined higher exposure versus the lowest exposure group (3 studies, 2,306 participants; OR_p_ = 1.40, 95% 1.14, 1.71; I^2^ = 0%; Fig. 4c). However, there was no evidence of an association between nitrate in drinking water and spina bifida when comparing the highest versus the lowest exposure groups (2 case–control studies, numbers of participants unknown; OR_p_ = 2.84, 95% CI 0.90, 8.97; I^2^ = 44.3%) or when comparing all combined higher versus the lowest nitrate exposure group (2 case–control studies, numbers of participants unknown; OR_p_ = 2.69, 95% CI (0.69, 10.49); I^2^ = 56.9%). There was also no association between nitrate in drinking water and anencephaly when comparing the highest to the lowest exposure groups (2 case–control studies, numbers of participants unknown; OR_p_ = 0.82, 95% CI 0.50, 1.36; I^2^ = 0.0%), or when comparing all combined higher versus the lowest nitrate exposure group (2 case–control studies, numbers of participants unknown; OR_p_ = 0.71, 95% CI 0.48, 1.05; I^2^ = 0.0%).

#### Any congenital heart defects

**Figure 5:**
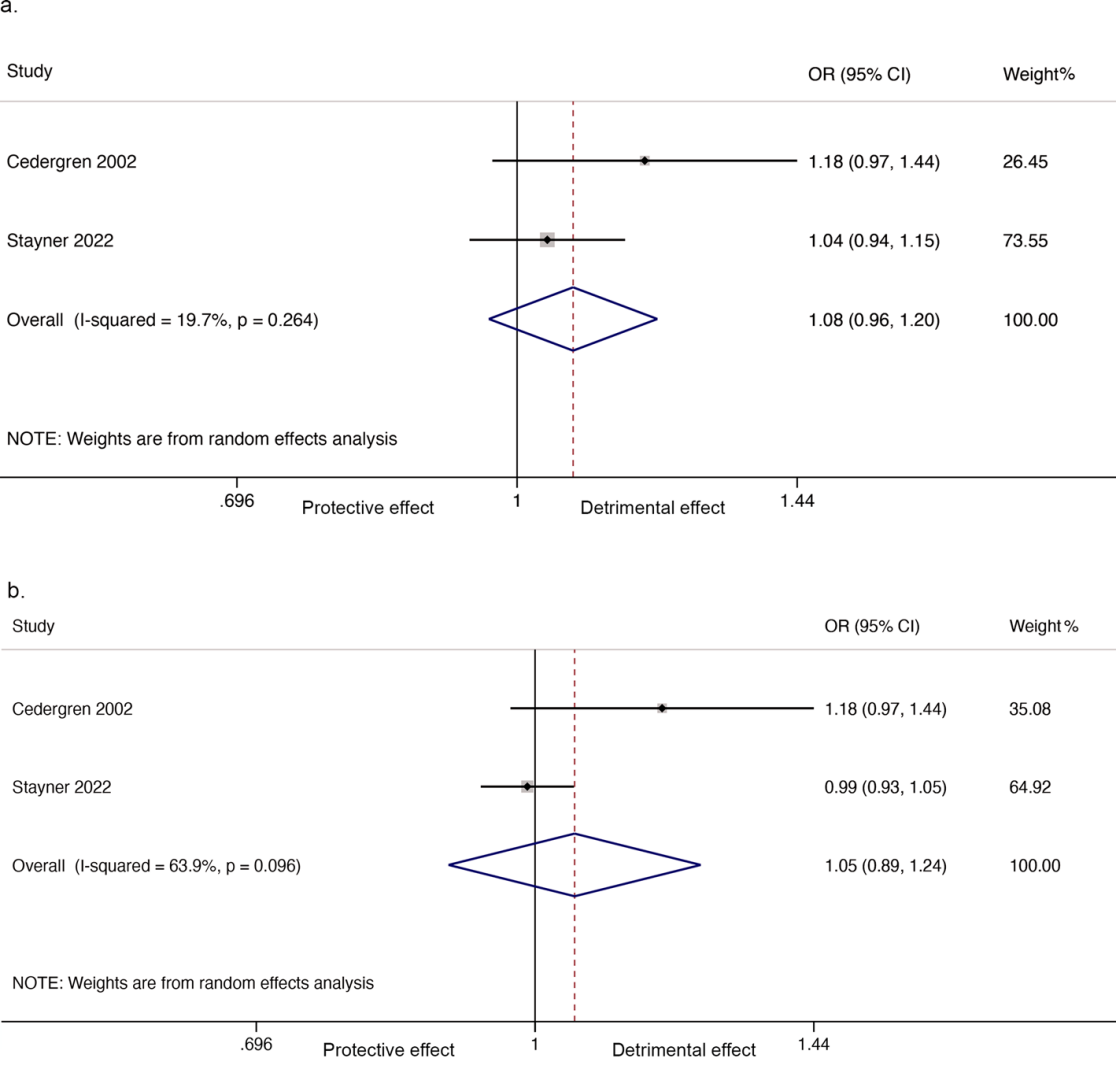
Forest plot for the association between nitrate in drinking water and any heart defects. (**a**) Highest versus the lowest exposure; (**b**) All combined higher versus the lowest exposure.

There were insufficient data to allow the linear relationship analysis. There was no evidence of an association between nitrate in drinking water and any congenital heart defects when comparing the highest versus the lowest nitrate exposure group (2 cohort studies, numbers of participants unknown; OR_p_ = 1.08, 95% CI 0.96, 1.20; I^2^ = 19.7%; Fig. 5a). However, when comparing all combined higher versus the lowest nitrate exposure group, there was significant between study heterogeneity (2 cohort studies, numbers of participants unknown; OR_p_ = 1.05, 95% CI 0.89, 1.24; I^2^ = 63.9%; Fig. 5b).

There were insufficient data to allow meta-analysis for any of the other outcomes. The direction of findings from the included studies are summarised in Table 3, with the detailed individual study results provided in Table S1.

**Table 3:** Direction of findings.

Outcome	Direction of association		
	Studies showing possible benefit	Studies showing no clear difference	Studies showing possible harm
**Spontaneous abortion**	Aschengrau 1989	Ebdrup 2022	–
**Stillbirth**	–	Thomesen 2021 Aschengrau 1993	–
**Neonatal death**	–	Aschengrau 1993	–
**Preterm birth**	–	Albouy-Llaty 2016 Liu 2008 Satyner 2017* Super 1981*	Coffman 2022 Bukowski 2001* Sherris 2021 Huang 2018*
**Prelabour rupture of membranes**	–	–	Joyce 2008*
**SGA**	–	Super 1981*	Migeot 2013
**LBW**	–	Coffman 2021 Liu 2008 Blake 2014* Stayner 2017*	Bukowski 2001*
**Birthweight**	–	–	Coffman 2021
**Length at birth**	–	–	Coffman 2021
**Head circumference at birth**	–	Coffman 2021	–
**Any birth defects**	Stayner 2022	Aschengrau 1993 Holtby 2014	Dorsch 1984 Ouattara 2022*
Limb deficiencies	–	Stayner 2022	Brender 2013 Blaisdel 2019*
Any oral clefts	–	Stayner 2022 Blaisdel 2019* Winchester 2009*	Brender 2013
Cleft lip without cleft palate	–	Liu 2008	Brender 2013
Cleft palate	–	Liu 2008	Brender 2013
Abdominal wall defect/ Gastroschisis	–	Stayner 2022 Waller 2010 Blaisdel 2019* Mattix 2007* Winchester 2009*	–
Digestive system defect	–	Stayner 2022	–
Ear, face and neck defect	–	Stayner 2022	–
Eye defect	–	–	Stayner 2022
Male genital defect	–	Stayner 2022	
Hypospadias	–	–	Blaisdel 2019*
Female genital defect	Stayner 2022	–	–
Respiratory defect	–	Stayner 2022 Winchester 2009*	
Urinary defect	Stayner 2022	–	–
**Any nervous system defects**	–	Arbuckle 1988 Stayner 2022	–
Any neural tube defects	–	Stayner 2022 Brender 2004 Croen 2001 Liu 2008 Blaisdel 2019*	Brender 2013
Spina bifida	–	Stayner 2022 Winchester 2009*	Brender 2004 Brender 2013
Anencephaly	–	Brender 2004 Brender 2013 Stayner 2022	–
Encephalocele	–	Stayner 2022	–
Hydrocephalus	–	Stayner 2022 Liu 2008	–
Microcephalus	–	Stayner 2022	–
**Any congenital heart defects**	–	Brender 2013 Stayner 2022 Blaisdel 2019*	Cedergren 2002
Conotruncal heart defects	–	Brender 2013	–
Patent ductus arteriosus	–	Liu 2008	–
Right ventricular outflow tract obstruction	–	Brender 2013	–
Left ventricular outflow tract obstruction	–	Brender 2013	–
Septal defects	–	Brender 2013	–
Atrial septal defects	–	Liu 2008	–
Ventricular septal defects	–	Liu 2008	–
Tetralogy of fallot	–	Liu 2008	–
**Down syndrome**	Liu 2008	Blaisdel 2019* Winchester 2009*	–

“–”: no study falls into this category. “*”: ecological, cross-sectional or prevalence study.

Aschengrau^43^ did not find an association between nitrate in drinking water and neonatal death. Thomsen^37^ and Aschengrau^42^ reported no association between nitrate in drinking water and stillbirth. Aschengrau^43^ found a decrease in the frequency of spontaneous abortion with any detectable level of nitrate. However, Ebdrup^33^, in a large cohort study, reported there was no association between drinking water nitrate and the risk of pregnancy loss. The continuous analysis indicated a risk of pregnancy loss in the lower nitrate exposure groups for the first trimester.

Migeot^38^ reported exposure to the second tertile (3.19–6.10 mg/L NO_3_-N) of nitrate compared with exposure to the lowest tertile (< 3.19 mg/L NO_3_-N) was associated with a possible increased risk of SGA birth (OR = 1.74, 95% CI 1.10, 2.75), but there was no association between exposure to the highest (> 6.10 mg/L NO_3_-N) and lowest tertiles of nitrate with SGA birth (OR = 1.51, 95% CI 0.96, 2.40)^38^.

Coffman^13^ and Liu^48^ reported no association between nitrate in drinking water and low birth weight, although compared to the lowest exposure group (≤ 0.23 mg/L NO_3_-N), Coffman^13^ reported that all the other exposure groups were associated with a small decrease in birth weight. They also reported that mean body length at birth decreased with increased nitrate in drinking water, but only in the second highest exposure group (1.13 to ≤ 5.65 mg/L NO_3_-N) not the highest exposure group (> 5.65 mg/L NO_3_-N). They reported no association between nitrate in drinking water and head circumference at birth.

Stayner^36^ reported there was no association between nitrate in drinking water and limb deficiencies or oral cleft defects. However, Brender^28^ reported that compared to the lowest tertile (< 0.71 mg/L), infants exposed to the highest tertile (> 3.5 mg/L NO_3_-N) of nitrate in drinking water had a potential increased risk of limb deficiencies and oral cleft defects.

Both a cohort study^36^ and a case–control study^49^ reported there was no association between nitrate in drinking water and abdominal wall defects/ gastroschisis.

### Evidence from other study types

Blake^50^ used a spatial analysis to explore the relationship between nitrate exposure level within ZIP codes and low birth weight. The authors reported there was no correlation between low birth weight and unsafe nitrate levels (> 10 mg/L as NO_3_-N). Two ecological studies^15,26^ of the same cohort explored the association between average county-level nitrate concentrations in drinking water and adverse birth outcomes. Antenatal exposure to nitrate in drinking water was associated with a possible increased risk of limb deficiencies, but no association was found between antenatal exposure to nitrate in drinking water and preterm birth, low birth weight, neural tube defects or oral cleft defects. However, Bukowski^51^ explored the association between residential postcode-level nitrate concentration in drinking water and low birth weight and preterm birth and reported that antenatal exposure to > 3.1 mg/L median nitrate concentration was associated with preterm birth and low birth weight. Mattix^52^ linked the monthly abdominal wall defect rates to monthly surface water nitrate concentration, and reported no association between nitrate levels in surface water and monthly abdominal wall defects rate. Winchester^53^ linked mean monthly nitrate concentration to birth defects and found there was no association between increased nitrate level (1.31 ± 0.20 vs. 0.16 ± 0.02 mg/L in log) in April–July (annual peaks in nitrates) and spina bifida, oral cleft, circulation system defects, Down syndrome, gastroschisis, urogenital defects and clubfoot or oral cleft. Ouattara^54^ assessed the occurrence of birth defects and nitrate concentrations collected from selected Nebraska watershed boundaries, and observed a positive association between high levels of nitrate (> 6.94 mg/L) in drinking water and the prevalence of birth defects with incidence rate ratio 1.44 (1.40–1.50). Two cross-sectional studies^55,56^ linked birth records, maternal and infant hospital discharge records to the CalEnviro Screen 3.0 dataset from California Communities Environmental Health Screening Tool to explore the relationship between preterm birth, gestational hypertension, eclampsia and environmental factors including nitrate in drinking water. The investigators reported that nitrate in drinking water is potentially associated with preterm birth in California. There was insufficient evidence suggesting that nitrate in drinking water was associated with hypertensive disorders or eclampsia in pregnancy. However, Super^57^ found there were no associations between well-water with high nitrate regions (> 4.518 mg/L as NO_3_-N) and preterm birth or size of infant at birth.

Joyce^58^ conducted a record-based prevalence study assigning water contaminant measurements to the maternal residential address. The authors found increasing exposure to nitrate in drinking water was associated with an increased risk of prelabor rupture of membranes (Table S2).

There were insufficient data to allow the sensitivity analyses or assessment of publication bias funnel plots.

## Discussion

In this systematic review of evidence from nine high-quality and seven moderate-quality observational studies, involving 7,268,991 participants, we found nitrate in drinking water may be associated with some adverse reproductive and birth outcomes. Studies included in the meta-analysis found an association between nitrate in drinking water and increased risk of preterm birth. Three high quality cohort studies contribute to this result. Coffman^35^ linked 1,009,189 liveborn resident in Denmark to household level of nitrate in drinking water. They observed an increasing risk of preterm birth with increases in nitrate in household tap water at levels below current regulatory levels. Sherris^34^, linked over 6 million birth certificate records to public water system monitoring records in US and suggested a robust association between nitrate in tap water and risk of preterm birth in within-mother analyses. They also found modestly increased odds of preterm birth within nitrate levels below the regulatory limit. Albouy-Llaty^40^ linked 13,654 mother/neonate pairs in France to mean nitrate concentrations in maternal place of residence and did not find a relationship between exposure to nitrate in drinking-water during the second trimester of pregnancy and preterm birth.

Our analysis from case–control studies also showed nitrate in drinking water may be associated with increased risk of neural tube defects, with a pooled odds ratio of 1.06 (1.02, 1.10) per 1 mg/L NO_3_-N increase in drinking water, although this finding is in contrast to a cohort study showing no linear association^36^. However, our analyses did not find a relationship between nitrate and any birth defects, or any congenital heart defects, although visual inspection of forest plots from meta-analyses shows mostly positive associations between nitrate and adverse reproductive and birth outcomes but with wide confidence intervals. It may therefore be possible that there is small impact of nitrate in drinking water on these outcomes, but the available studies together are insufficiently powered to detect a clinically significant effect.

We observed moderate heterogeneity in some of our meta-analyses. Several factors may contribute to the inconsistent findings amongst studies. The first factor is the different study characteristics. In the analysis of preterm birth, for example, Albouy-Llaty 2016 was conducted in France with a reported preterm birth rate of 7.5% of all live births in 2016^59^, while Sherris 2021 was conducted in the US with a reported preterm birth rate of 10.1% of all live births in 2020^60^, and Coffman 2022 was conducted in Demark with a reported preterm birth rate of 6.29% per year between 2016 and 2020^61^. In addition, the three studies monitored different exposure periods: Albouy-Llaty 2016 estimated the nitrate exposure during the second trimester of pregnancy, while Coffman 2022 and Sherris 2021 measured the nitrate exposure throughout pregnancy. Moreover, while Albouy-Llaty 2016 studied 13,481 participants, only 4625 participants were included in their adjusted analytical model, so that statistical power was very limited. In comparison, Coffman 2022 analyzed data from 1,009,189 participants and Sherris 2021 analyzed data from 3,832,090 participants. Another factor may be the different statistical models used and adjustment for different confounding factors. Albouy-Llaty 2016 used a multivariable logistic regression model adjusted for rural area, season, maternal age, mother’s occupation, smoking during pregnancy, single-parent family, history of preterm birth, primiparity and quality of follow-up; Coffman 2022 used generalized logistic regression models adjusted for the non-independence of births from the same mother and year, sex, gravidity, urbanicity, and maternal age, smoking, education, income, and employment status; Sherris 2021 used mixed-effects logistic regression adjusted for maternal age, parity, education, race, payer for delivery, and timing of initiation of care. Therefore, without individual participant data we could not further investigate nonlinear effects.

Further, the limits of exposure categories were different between studies. To calculate the exposure–response relationship for nitrate in drinking water, our study used several transformations. For the study by Albouy-Llaty 2016, when adjusting for confounders, both relationships between preterm birth and second tertile nitrate exposure group, and preterm birth and third tertile nitrate exposure group were non-significant, but the direction of the findings was towards a protective effect. Therefore, the result of the generalized least squares regression analysis yielded negative study-specific slopes and the odds ratio for 1 mg/L increase in nitrate indicated a protective effect. For the study by Sherris 2021, the odds ratio for 1 mg/L was reported by the authors but for different gestational age subgroups (20–31 gestational weeks and 32–36 gestational weeks). The study by Coffman 2022 reported adjusted odds ratio for per 10 mg/L NO_3_^−^. We pooled the odds ratios for all these subgroups to obtain the results for 1 mg/L increase in nitrate for overall preterm birth. This pooled estimate indicates nitrate in drinking could be a risk factor for preterm birth, but the several steps of conversion may reduce the accuracy of the estimation.

Periconceptional intake of folic acid reduces the risk of women having an infant affected by neural tube defects^62^. The inconsistent findings for neural tube defects from cohort^36^ and case–control^28,44,45^ studies may result from the different years of outcome ascertainment as it is likely more pregnant women received folic acid in the later studies. However, Stayner 2022 included all singleton infants born in Denmark between 1991 and 2013, where periconceptional folic acid has been recommended since 1997^63^. The three case–control studies included infants born in U.S. between 1989 and 1991, 1995–2000 or 1997–2005. The U.S. recommended periconceptional intake of folic acid in 1992^64^ and implemented mandatory fortification of cereal grains with folic acid in 1998^65^.

The nitrate levels in drinking water reported in the different studies vary, but most of the high-quality studies investigated levels that were within the WHO guideline nitrate level (11.3 mg/L NO_3_-N). However, Sherris, who reported a relationship between high nitrate exposure and preterm birth^14^ selected the high exposure cut-off as > 10 mg/L NO_3_-N and median exposure cut-off as 5 mg/L NO_3_-N to correspond to MCL level and half MCL level respectively for nitrate in drinking water in US, although only 0.6% of the population were exposed to this level. Further, the ecological study by Blake et al.^50^, which did not find an association between preterm birth and unsafe nitrate level in drinking water, also assessed exposure to the unsafe nitrate level in drinking water of 10 mg/L NO_3_-N. The maximum nitrate level in drinking water (19.3 mg/L NO_3_-N) reported in the two cross-sectional studies^55,56^ was almost equivalent to twice the US MCL of 10 mg/L NO_3_-N, but 75% of the population in the study region were exposed to < 2.44 mg/L NO_3_-N. Croen^45^ found exposure to the highest nitrate level, which was above the US MCL, was associated with increased risk for anencephaly, but only 3% of the included population were exposed to this level.

Some drinking water is pumped from the ground, other drinking water originates as surface water in streams and rivers. About half of New Zealand’s drinking water is pumped from the ground, with the remainder coming from surface sources^66^. In the US, more water systems use groundwater (78%) rather than surface water as a source, but more people (68%) receive their water from a system supplied by surface water^67^.

Groundwater is recharged from the surface, predominantly from rainfall, but can also receive leakage from rivers and lakes^4^. Drinking water suppliers in New Zealand are not required to routinely monitor or report on nitrate levels if levels have been previously found to be below 25 mg/L as NO_3_^−^^68^. Richard^69^ estimated the variability of nitrate levels in drinking water in New Zealand, and found the nitrate levels in drinking water from registered supplies ranged from less than detection (< 0.01 mg/L) to 41.8 mg/L as NO_3_^−^. More than 60% of the population were exposed to less than 2 mg/L as NO_3_^−^, 8.2% of the population were exposed to more than 5 mg/L as NO_3_^−^, 2.2% were exposed to more than 10 mg/L as NO_3_^−^, and 0.1% of the population were exposed to more than 25 mg/L as NO_3_^−^^69^. Although the nitrate level in groundwater in New Zealand is lower than the WHO guideline level, long-term trends (10 years, 2009–2018) showed 28–35% of sites had increasing levels of nitrate over time^70^. Further, nitrate contamination present in the groundwater would likely stay there for years or decades, so exposures identified are likely to continue to increase if nitrate removal technologies are not utilized^71^.

A recent report estimated that while New Zealanders have similar nitrate exposure from drinking-water to that in most other countries, total nitrate intake from drinking water is less than 10%^3^. While this is of note, the conclusions drawn in the report should be interpreted with caution as the data analyzed were from more than ten years ago and the outcome of interest was limited to the risk of colorectal cancer rather than adverse perinatal outcomes. As nitrate concentrations in drinking water increase, it will contribute a larger proportion of total nitrate intake, potentially making this exposure of greater importance for overall public and reproductive health.

High quality, large epidemiology studies are needed to further assess any associations with perinatal outcomes and nitrate exposure from drinking water. In the US, all public water systems are required to be monitored at least annually to determine compliance with the nitrate MCL of 10 mg/L as NO_3_-N^72^. However, nitrate concentrations in New Zealand are not regularly monitored if below 50% of MAV^68^, and there is no national repository of nitrate exposure data for the New Zealand population^69^. In the recently published high quality studies, Sherris 2021 included around 6 million participants and Coffman 2021 included 852,348 participants. In New Zealand, there are around 58,000 births annually and the estimated preterm birth rate is 7.4%^73^. Thus, it will be difficult to reach adequate sample size to draw reliable conclusions about nitrate exposure and reproductive outcomes, since this would require matching at least 15 years of birth data with individual or regional nitrate exposure in drinking water. This issue of very limited power to detect small effect sizes is a problem in many other smaller regions, making it difficult to investigate the effects of nitrates in local drinking water supplies.

Two previous systematic reviews have assessed the association between maternal nitrate intake and risk of neural tube defects^30^, and between maternal intake of nitrate and risk of birth defects and preterm birth^31^. They reported no association between maternal nitrate intake and the risk of preterm birth, limb deficiency, cleft lip, and neural tube defects through high versus low meta-analysis. However, non-linear analysis showed the risk of neural tube defects increased with increasing intake of maternal dietary nitrate > 3 mg/day, and there was positive correlation between nitrate intake and heart defects through high versus low meta-analysis, with each additional 0.5 mg/day of maternal nitrate intake increasing the risk of heart defects. These two systematic reviews assessed maternal dietary nitrate intake, including nitrate from drinking water, food, or drugs. However, there was some overlapping of participants between the included studies that used the same cohort, with some participants counted more than once, potentially resulting in overestimation of the effects.

This review has some limitations. First, most of the studies were carried out in US and Europe. Given that nitrate levels in drinking water vary widely among different regions and countries, findings from these studies should be interpreted with caution when extrapolated to other regions. Second, the studies included in this systematic review do not consistently account for other potential confounding factors such as maternal diet, nitrosatable drug use, and antioxidant intake. Third, the concentration of nitrate in water often fluctuates with the season^1^. Not all studies included in this review took seasonal variation of nitrate into account in the measurement of exposure or as a factor in the adjusted model. Nitrogen shows significant seasonal relationships with high-intensity agriculture, with the difference between summer and winter water quality increasing as the proportion of high intensity agriculture in a catchment increases. For example, spatial modelling in New Zealand showed that regions dominated by high-intensity agriculture typically have poorer clarity, turbidity and nutrient concentrations in winter than in summer^74^. Fourth, in meta-analysis of observational studies, it is challenging or impossible to identify any unpublished studies, as pre-registration of a protocol is not mandatory^75^. Finally, of all the pre-specified outcomes, only a few outcomes could be incorporated into a meta-analysis to help determine the overall association. Sixteen studies have evaluated the nitrate in drinking water and adverse reproductive and birth outcomes, but the number of studies of any individual outcome was limited. Although some outcomes were reported by more than one study, studies using different designs cannot be combined.

We conclude that currently there is sufficient evidence of a possible association between nitrate in drinking water and preterm birth and specific congenital anomalies, to warrant nitrate exposure monitoring and reporting, and regular review as new evidence becomes available.

## Methods

The study was reported according to the PRISMA guidelines (Note S1)^76^. The review protocol was not registered but was prepared before the review was conducted (Note S2).

### Criteria for considering studies for this review

Type of studies: randomized trials, cohort and case–control studies published in English that reported the relationship between nitrate intake from drinking water and the risk of perinatal outcomes were eligible.

Type of participants: pregnant women and their infants.

Type of intervention: the exposure of interest is nitrate intake from drinking water during the antenatal period.

Type of outcome measure:

Primary outcome: a composite of any of the following outcomes: preterm birth; small-for-gestational-age (SGA) infant; low birth weight infant; miscarriage; stillbirth; and neonatal death.

Secondary outcomes: For infants: preterm birth, SGA, low birth weight, stillbirth, neonatal death, perinatal death, hypoglycemia, need for respiratory support after birth, infection, congenital abnormality, necrotizing enterocolitis, bronchopulmonary dysplasia, intraventricular hemorrhage, neonatal lung disease, neonatal intensive care unit (NICU) admission, jaundice, methemoglobinemia (as defined by the authors).

For women: any pregnancy complications (miscarriage, high blood pressure, preeclampsia, gestational diabetes, infection, obstetric hemorrhage; as defined by the authors).

### Search strategy

We conducted a comprehensive search of databases from inception to 30 November 2022, including: Ovid MEDLINE via PubMed, Embase, CINAHL, Cochrane Central Register of Controlled Trials (CENTRAL, current issue) in the Cochrane Library, Web of Science, Scopus, GEOBASE and ProQuest Agricultural and Environmental Science Database, using search terms unique to the review topic (Note S3). We searched using both English and American spelling. We did not apply language restrictions, but only full text articles in English were included. Additionally, we reviewed the reference lists of all identified articles for relevant articles not identified in the primary search.

### Study selection

Two authors (LL and SSC) independently evaluated and appraised the retrieved studies using COVIDENCE, extracted data and assessed risk of bias. Any disagreements were resolved by discussion and, if necessary, by discussion with a third review author (JH).

Selection of studies followed the steps below:

1. Import all the records from the database into COVIDENCE (https://www.covidence.org/).
2. Screen titles and abstracts to select relevant reports and exclude studies not relevant for this review.
3. Examine full-text studies for compliance with the eligibility criteria for this review.
4. Make final decisions on study inclusion and proceeded to data collection.

### Data extraction

We recorded the selection process in sufficient detail to complete a PRISMA flow diagram.

We developed a data form (Note S4) to extract data for eligible studies. Information extracted included: source details, eligibility assessment, methodological details, characteristics of participants, details of intervention and outcomes reported.

### Study quality

We assessed the quality of case–control and cohort studies according to the Newcastle–Ottawa Scale (NOS)^77^. The NOS evaluates nine methodological items and their reporting (participant selection, comparability of groups, and ascertainment of exposure/outcome), with values ≥ 7 compatible with good study quality (least bias, results are considered valid), between 2 and 7 with moderate study quality (susceptible to some bias but probably not enough to invalidate the results), and ≤ 2 with poor study quality (significant bias that may invalidate results).

We planned to assess the quality of randomized trials using the methods specified in the Cochrane Handbook for Systematic Reviews of Interventions^78^: (1) random sequence generation (selection bias); (2) allocation concealment (selection bias); (3) blinding of participants, personnel and outcome assessment (performance and detection bias); (4) incomplete outcome data (attrition bias); (5) selective reporting (reporting bias); (6) other bias (checking for bias due to problems not covered by (1) to (5) above).

### Statistical analysis

The nitrate values in the analyses are expressed as nitrate-nitrogen (NO_3_-N). We converted the nitrate (NO_3_^−^ mg/L) values of the included studies to nitrate-nitrogen (NO_3_-N mg/L) by multiplying 0.2259^1^.

The relationships between nitrate intake from drinking water and the risk of adverse birth outcomes were examined based on the effect size. Nitrate intake from drinking water, odds ratios (ORs), risk ratios (RRs), hazard ratios (HRs), with 95% confidence intervals (CIs) were extracted (both crude and adjusted).

Because different studies used different exposure categories and have presented data in a variety of ways, we pooled the study-specific risk per mg/L increase in nitrate for each outcome, using the mid-point of each reported exposure category. If the lower and upper limits of the category were given, the midpoint intake of nitrate in drinking water was calculated as: midpoint intake = (lower limit + upper limit) divided by 2. If the midpoint intake was given, the data were used directly. If the interval for any category of nitrate intake was not provided, we assigned a value following the algorithms suggested by Il'yasova et al.^79^. For the upper open-ended category, we assigned the value of its lower limit plus the width of the previous (second-to-highest) interval. For the lower open-ended category, we assigned the value of its upper limit minus half the width of the next (second-to-lowest) interval. If the range of lower open-ended category was smaller than the half width of the next (second-to-lowest) interval, we assigned the value of half of the upper limit.

Generalized least squares regression analysis was used to generate study-specific slopes representing the estimated increase in log odds ratio (OR) per mg/L increase in drinking water nitrate concentration and standard errors for these slopes. Study-specific slopes and their standard errors were then used to calculated ORs and 95% confidence intervals (CIs) per mg/L increase in nitrate for each outcome. When the OR per mg/L was given, the data were used directly. We then incorporated the OR per mg/L into meta-analysis using a random effects model to derive a weighted pooled estimate with 95% CIs based on the DerSimonian and Laird method^80^.

We also calculate the pooled ORs for the highest compared with the lowest exposure level, and for all combined exposure levels above the lowest compared with the lowest exposure level using random effects models.

A random effects model was used instead of a fixed effects model in order to account for both within-study and inter-study variation. Heterogeneity tests were performed using the I-square and Q-statistic, and significant heterogeneity was defined as I^2^ > 50% or p < 0.10^78^. We planned to assess potential bias due to small study effects by visual inspection of funnel plots when there were more than 10 studies. We planned to conduct sensitivity analyses by examining only studies considered to be of good quality.

Statistical analyses were performed using Stata Statistical Software (version 14, STATA).

## Supplementary Information

Supplementary Information.

## Data Availability

Metadata, along with instructions for data access, are available at the University of Auckland’s research data repository, Figshare (https://auckland.figshare.com). Data access requests are to be submitted to the Data Access Committee via researchhub@auckland.ac.nz. Data will be shared with researchers who provide a methodologically sound proposal and have appropriate ethical and institutional approval. Researchers must sign and adhere to the Data Access Agreement that includes a commitment to using the data only for the specified proposal, to store data securely and to destroy or return the data after completion of the project. The Data Access Committee reserves the right to charge a fee to cover the costs of making data available if required.
