# Supplementary material for: Nitrate contamination in drinking water and adverse reproductive and birth outcomes: a systematic review and meta-analysis
Source: Sci Rep. 2023 Jan 11;13:563. doi: 10.1038/s41598-022-27345-x (PMC9834225; doi:10.1038/s41598-022-27345-x)
Supplement: Supplementary file 1 — Supplementary Information. [file 41598_2022_27345_MOESM1_ESM.pdf]

SUPPLEMENTARY INFORMATION

Nitrate contamination in drinking water and adverse reproductive and birth outcomes: a systematic review and meta-analysis

Luling Lin[1], Sophie St Clair[1], Greg D Gamble[1], Caroline A Crowther[1], Lesley Dixon[2], Frank H Bloomfield[1], *Jane E Harding[1]

[1]Liggins Institute, University of Auckland, Auckland, New Zealand

[2] New Zealand College of Midwives, 376 Manchester Street, Richmond, Christchurch 8014, New Zealand

Correspondence: E-mail: j.harding@auckland.ac.nz, Telephone: +6499236439

Supplement Table S1. Outcomes from included studies.

Outcome	Study	Study design	Case	Controls	Total	Exposure groups NO_3-N (mg/L)	Unadjusted OR/MD (95%CI)	Adjusted OR/MD (95% CI)
Spontaneous abortion	Aschengrau 1989	Case-control	-	-	-	Undetectable	1	1
			-	-	-	0.1 to 5.5	0.4 (0.3, 0.6)	0.5 (0.2, 0.9)
Spontaneous pregnancy loss before 22 weeks completed gestation	Ebdrup 2022	Cohort	831	23,998	24,829	≤0.23	-	1
			829	22,645	23,474	>0.23 to ≤0.45	-	0.97 (0.88-1.07)
			781	21,232	22,013	>0.45 to ≤1.13	-	1.00 (0.90-1.10)
			346	9,240	9,586	>1.13 to ≤5.65	-	1.04 (0.92-1.18)
			128	3,545	3,673	>5.65	-	0.97 (0.81-1.17)
Stillbirth	Thomsen 2021	Cohort	616	151,433	152, 049	≤0.23	1	1
			713	198,781	199,494	>0.23 to ≤0.45	0.88 (0.79, 0.98)	0.97 (0.80, 1.18)
			749	214,620	215,369	>0.45 to ≤1.13	0.86 (0.78, 0.96)	0.88 (0.72, 1.07)
			240	63,221	63,464	>1.13 to ≤5.65	0.93 (0.80, 1.08)	0.95 (0.73, 1.24)
			93	22,344	22,437	>5.65	1.01 (0.81, 1.26)	1.26 (0.89, 1.79)
	Aschengrau 1993	Case-control	77	1,177	1,254	Undetectable to 0.1	1	-
						0.2	1.0 (not significant)	-
						0.3 to 4.5	0.8 (not significant)	-
Neonatal death		Case-control	55	1177	1,232	Undetectable to 0.1	1	1

Outcome	Study	Study design	Case	Controls	Total	Exposure groups NO_3-N (mg/L)	Unadjusted OR/MD (95%CI)	Adjusted OR/MD (95% CI)
	Aschengrau 1993					0.2	1.7 (not significant)	2.0 (not significant)
						0.3 to 4.5	1.3 (not significant)	1.2 (not significant)
Preterm birth	Albouy-Llaty 2016	Cohort	186	4,307	4,493	< 3.64	1	1
			209	4,299	4,508	3.64 to 6.14	1.17 (0.92, 1.38)	0.89 (0.55, 1.43) [b]
			187	4,293	4,480	> 6.14	1.01 (0.82, 1.24)	0.75 (0.46, 1.23) [b]
	Coffman 2022	Cohort	26,616	502,556	529,172	< 0.45	-	1
			16,547	301,588	318,135	0.45 to <1.13	-	1.03 (1.01-1.06)
			6,579	118,023	124,602	1.13 to <5.65	-	1.04 (1.01-1.07)
			2,005	35,275	37,280	≥ 5.65	-	1.05 (1.00-1.10)
			51,747	957,442	1,009,189	Continuous	-	1.01 (1.00-1.03)
	Sherris 2021	Cohort	148,599	3,404,894	3,553,493	< 5.0	-	1
			18,946	404,732	423,678	5 to < 10	-	1.01 (1.009, 1.013) [c]
			966	22,464	23,430	≥ 10	-	1.003 (1.002, 1.004) [c]
	Liu 2008	Case-control	-	-	-	Continuous	1.02 (0.96, 1.08)	Not significant
SGA	Migeot 2013	Cohort	120	1,642	1,762	< 3.1	1	1
			281	2,835	3,116	3.19 to 6.10	1.40 (1.12, 1.74)	1.74 (1.10, 2.75)
			257	2,739	2,996	> 6.10	1.29 (1.03, 1.63)	1.51 (0.96, 2.40)

Outcome	Study	Study design	Case	Controls	Total	Exposure groups NO_3-N (mg/L)	Unadjusted OR/MD (95%CI)	Adjusted OR/MD (95% CI)
Low birth weight	Coffman 2021	Cohort	2,026	184,156	186,182	≤ 0.23	-	1
			2,057	180,813	182,870	0.23 to ≤ 0.45	-	0.98 (0.92, 1.05)
			3,573	295,895	299,468	0.45 to ≤ 1.13	-	1.01 (0.94, 1.08)
			1,972	148,047	150,019	1.13 to ≤ 5.65	-	1.02 (0.95, 1.09)
			400	33,409	33,809	> 5.65	-	0.99 (0.88, 1.12)
	Liu 2008	Case-control	-	-	-	continuous	0.98 (0.93, 1.04)	Not significant
Birth weight (g)	Coffman 2021	Cohort	-	-	186,182	≤ 0.23	-	0
			-	-	182,870	0.23 to ≤ 0.45	-	-3.6 (-6.8, -0.5)
			-	-	299,468	0.45 to ≤ 1.13	-	-7.4 (-10.8, -4.1)
			-	-	150,019	1.13 to ≤ 5.65	-	-8.1 (-11.6, -4.6)
			-	-	33,809	> 5.65	-	-7.0 (-13.3, -0.7)
Body length at birth (mm)	Coffman 2021	Cohort	-	-	185,379	≤ 0.23	-	0
			-	-	182,001	0.23 to ≤ 0.45	-	-0.1 (-0.2, 0.1)
			-	-	297,885	0.45 to ≤ 1.13	-	-0.2 (-0.3, -0.02)
			-	-	149,114	1.13 to ≤ 5.65	-	-0.4 (-0.5, -0.2)
			-	-	33,727	> 5.65	-	-0.2 (-0.5, 0.1)
	Coffman 2021	Cohort	-	-	140,486	≤ 0.23	-	0

Outcome	Study	Study design	Case	Controls	Total	Exposure groups NO_3-N (mg/L)	Unadjusted OR/MD (95%CI)	Adjusted OR/MD (95% CI)
Head circumference at birth (mm)			-	-	126,561	0.23 to ≤ 0.4	-	0.02 (-0.1, 0.2)
			-	-	218,398	0.45 to ≤ 1.13	-	-0.2 (-0.4, -0.1)
			-	-	81,085	1.13 to ≤ 5.65	-	0.1 (-0.1, 0.2)
			-	-	22,451	> 5.65	-	0.1 (-0.2, 0.3)
Any birth defects	Stayner 2022	Cohort	17,264	510,195	527,459	< 0.45	1	1
			10,653	310,986	321,639	0.45 to <1.13	1.01 (0.99, 1.04)	1.01 (0.98, 1.04)
			4,092	126,645	130,737	1.13 to <5.65	0.95 (0.92, 0.99)	0.99 (0.95, 1.02)
			1,173	37,906	39,079	≥ 5.65	0.91 (0.86, 0.97)	0.93 (0.88, 0.99)
			33,182	985,732	1,018,914	Continuous	0.97 (0.96, 0.99)	0.98 (0.97, 1.00)
	Aschengrau 1993	Case-control	1,039	1,177	2,216	Undetectable to 0.1	1	1
						0.2	0.9 (not significant)	1.0 (not significant)
						0.3 to 4.5	0.8 (not significant)	0.9 (not significant)
	Dorsch 1984	Case-control	70	107	177	< 1.13	-	1
			138	106	244	1.13 to 3.39	-	2.6 (1.6, 4.1)
			10	5	15	> 3.39	-	4.1 (1.3, 13.1)
	Holtby 2014	Case-control	127	353	480	< 1.0	-	1
			351	931	1,282	1.0 to 5.56	-	1.65 (0.83, 3.27)

Outcome	Study	Study design	Case	Controls	Total	Exposure groups NO_3-N (mg/L)	Unadjusted OR/MD (95%CI)	Adjusted OR/MD (95% CI)
			127	351	478	> 5.56	-	1.66 (0.81, 3.42)
Limb deficiencies	Stayner 2022	Cohort	5,528	-	-	< 0.45	1	1
				-	-	0.45 to <1.13	0.94 (0.90, 0.98)	1.02 (0.96, 1.08)
				-	-	1.13 to <5.65	0.98 (0.92, 1.04)	0.97 (0.89, 1.06)
				-	-	≥ 5.65	1.02 (0.92, 1.13)	0.91 (0.78, 1.06)
				-	-	Continuous	1.00 (0.96, 1.03)	0.99 (0.95, 1.03)
	Brender 2013	Case-control	23	370	393	< 0.71	1	1
			29	367	396	0.71 to 3.86	1.27 (0.72, 2.24)	1.17 (0.66, 2.07)
			42	368	410	> 3.86	1.84 (1.08, 3.11)	1.79 (1.05, 3.08)
Any oral cleft defects	Stayner 2022	Cohort	2,154	-	-	< 0.45	1	1
				-	-	0.45 to <1.13	1.07 (0.97, 1.17)	1.07 (0.97, 1.17)
				-	-	1.13 to <5.65	1.00 (0.88, 1.14)	0.97 (0.85, 1.11)
				-	-	≥ 5.65	0.97 (0.77, 1.23)	0.95 (0.76, 1.20)
				-	-	Continuous	0.99 (0.93, 1.05)	0.98 (0.92, 1.04)
	Brender 2013	Case-control	122	370	492	< 0.71	1	1
			120	366	486	0.71 to 3.86	0.99 (0.74, 1.33)	0.98 (0.73, 1.32)
			173	367	540	> 3.86	1.43 (1.09, 1.88)	1.45 (1.10, 1.92)

Outcome	Study	Study design	Case	Controls	Total	Exposure groups NO_3-N (mg/L)	Unadjusted OR/MD (95%CI)	Adjusted OR/MD (95% CI)
Cleft lip without cleft palate	Brender 2013	Case-control	24	370	394	< 0.71	1	1
			29	366	395	0.71 to 3.86	1.22 (0.70, 2.14)	1.13 (0.64, 1.99)
			47	367	414	> 3.86	1.97 (1.18, 3.30)	1.82 (1.08, 3.07)
	Liu 2008	Case-control	-	-	-	Continuous	0.92 (0.75, 1.12)	Not significant
Cleft palate	Brender 2013	Case-control	23	370	393	< 0.71	1	1
			29	366	395	0.71 to 3.86	1.12 (0.66, 1.88)	1.12 (0.66, 1.90)
			42	367	409	> 3.86	1.88 (1.17, 3.01)	1.90 (1.17, 3.09)
	Liu 2008	Case-control	-	-	-	Continuous	1.05 (0.85, 1.28)	Not significant
Abdominal wall defects / Gastroschisis	Stayner 2022	Cohort	445	-	-	< 0.45	1	1
				-	-	0.45 to <1.13	1.01 (0.82 1.25)	0.92 (0.74, 1.14)
				-	-	1.13 to <5.65	1.13 (0.86, 1.50)	0.87 (0.66, 1.16)
				-	-	≥ 5.65	1.21 (0.76, 1.90)	1.01 (0.64, 1.59)
				--	-	Continuous	1.08 (0.96, 1.21)	1.00 (0.88, 1.13)
	Waller 2010	Case-control	805	3,616	4,421	> 10	-	-
Digestive system defects	Stayner 2022	Cohort	2,139	-	-	< 0.45	1	1
				-	-	0.45 to <1.13	0.98 (0.88, 1.10)	0.96 (0.87, 1.06)
				-	-	1.13 to <5.65	0.93 (0.80, 1.08)	1.02 (0.89, 1.16)

**Outcome**	**Study**	**Study design**	**Case**	**Controls**	**Total**	**Exposure groups NO_3-N (mg/L)**	**Unadjusted OR/MD (95%CI)**	**Adjusted OR/MD (95% CI)**
				-	-	≥ 5.65	0.75 (0.56, 1.00)	0.70 (0.54, 0.92)
				--	-	Continuous	0.90 (0.85, 0.97)	1.03 (0.91, 1.17)
Ear, face, and neck defects	Stayner 2022	Cohort	448	-	-	< 0.45	1	1
				-	-	0.45 to <1.13	1.22 (0.99, 1.50)	0.96 (0.87, 1.06)
				-	-	1.13 to <5.65	1.04 (0.77, 1.39)	1.02 (0.89, 1.16)
				-	-	≥ 5.65	1.26 (0.80, 2.00)	0.70 (0.54, 0.92)
				-	-	Continuous	1.02 (0.90, 1.15)	1.03 (0.91, 1.17)
Eye defect	Stayner 2022	Cohort	1,402	-	-	< 0.45	1	1
				-	-	0.45 to <1.13	1.22 (0.99, 1.50)	1.02 (0.91, 1.16)
				-	-	1.13 to <5.65	1.04 (0.77, 1.39)	1.23 (1.05, 1.44)
				-	-	≥ 5.65	1.26 (0.80, 2.00)	1.29 (1.00, 1.66)
				-	-	Continuous	1.08 (1.01, 1.15)	1.09 (1.03, 1.16)
Male genital defect	Stayner 2022	Cohort	3,103	-	-	< 0.45	1	1
				-	-	0.45 to <1.13	1.10 (1.04, 1.18)	1.09 (1.02, 1.16)
				-	-	1.13 to <5.65	1.02 (0.93, 1.12)	0.99 (0.91, 1.09)
				-	-	≥ 5.65	0.88 (0.75, 1.03)	0.86 (0.74, 1.02)
				-	-	Continuous	0.97 (0.93, 1.01)	0.96 (0.92, 1.00)

Outcome	Study	Study design	Case	Controls	Total	Exposure groups NO_3-N (mg/L)	Unadjusted OR/MD (95%CI)	Adjusted OR/MD (95% CI)
Female genital defects	Stayner 2022	Cohort	203	-	-	< 0.45	1	1
				-	-	0.45 to <1.13	1.16 (0.90, 0.98)	1.12 (1.06, 1.20)
				-	-	1.13 to <5.65	1.11 (0.92, 1.04)	0.99 (0.91, 1.08)
				-	-	≥ 5.65	-	-
				-	-	Continuous	0.97 (0.98, 1.03)	0.94 (0.90, 0.98)
Respiratory defect	Stayner 2022	Cohort	1,287	-	-	< 0.45	1	1
				-	-	0.45 to <1.13	0.97 (0.90, 1.05)	1.22 (1.08, 1.38)
				-	-	1.13 to <5.65	0.85 (0.76, 0.95)	1.15 (0.97, 1.37)
				-	-	≥ 5.65	0.66 (0.53, 0.81)	0.85 (0.61, 1.18)
				-	-	Continuous	0.94 (0.87, 1.02)	0.95 (0.88, 1.03)
Urinary defect	Stayner 2022	Cohort	3,055	-	-	< 0.45	1	1
				-	-	0.45 to <1.13	0.97 (0.90, 1.05)	0.96 (0.89,1.04)
				-	-	1.13 to <5.65	0.85 (0.76, 0.95)	0.88 (0.79, 0.98)
				-	-	≥ 5.65	0.66 (0.53, 0.81)	0.67 (0.54, 0.83)
				-	-	Continuous	0.89 (0.84, 0.94)	0.90 (0.85, 0.95)
Any nervous system defect	Stayner 2022	Cohort study	1,702	-	-	< 0.45	1	1
				-	-	0.45 to <1.13	0.98 (0.88, 1.10)	0.98 (0.88,1.10)

**Outcome**	**Study**	**Study design**	**Case**	**Controls**	**Total**	**Exposure groups NO_3-N (mg/L)**	**Unadjusted OR/MD (95%CI)**	**Adjusted OR/MD (95% CI)**
				-	-	1.13 to <5.65	0.93 (0.80, 1.08)	0.93 (0.80, 1.09)
				-	-	≥ 5.65	0.75 (0.56, 1.00)	0.75 (0.57, 1.00)
				-	-	Continuous	0.94 (0.87,1.01)	0.94 (0.87, 1.01)
Central nervous system defects	Arbuckle 1988	Case-control	-	-	-	0.023	-	1
			-	-	-	0.081	-	0.93 (0.85, 1.00)
			-	-	-	0.088	-	0.92 (0.84, 1.00)
			-	-	-	0.734	-	0.39 (0.15, 1.05)
Any neural tube defects	Stayner 2022	Cohort	424	-	-	< 0.45	-	1
				-	-	0.45 to <1.13	-	0.89 (0.72, 1.10)
				-	-	1.13 to <5.65	-	0.72 (0.52, 1.00)
				-	-	≥ 5.65	-	0.88 (0.53, 1.46)
				-	-	Continuous	-	0.95 (0.82, 1.10)
	Brender 2004	Case-control	43	67	110	< 3.52	-	1
						≥ 3.52	-	1.9 (0.8, 4.6)
	Brender 2013	Case-control	67	367	434	< 0.65	1	1
			65	360	425	0.65 to 3.5	0.99 (0.68, 1.43)	1.00 (0.68, 1.45)
			95	374	469	> 3.5	1.39 (0.99, 1.96)	1.43 (1.01, 2.04)

Outcome	Study	Study design	Case	Controls	Total	Exposure groups NO_3-N (mg/L)	Unadjusted OR/MD (95%CI)	Adjusted OR/MD (95% CI)
	Croen 2001	Case-control	213	248	461	< 1.13	1	1
			101	87	188	1.13 to 3.39[a]	1.3 (0.96, 1.90)	1.3 (0.90, 2.0)
			106	86	192	3.61 to 7.91[a]	1.4 (1.0, 2.0)	1.6 (1.1, 2.3)
			16	11	27	8.13 to 15.14[a]	1.7 (0.78, 3.70)	1.7 (0.76, 4.0)
	Ericson 1988	Case-control	145	280	425	-	-	-
	Liu 2008	Case-control	-	-	-	Continuous	0.81 (0.62, 1.06)	Not significant
Spina bifida	Stayner 2022	Cohort	356	-	-	< 0.45	-	1
				-	-	0.45 to <1.13	-	0.90 (0.71, 1.13)
				-	-	1.13 to <5.65	-	0.78 (0.55, 1.09)
				-	-	≥ 5.65	-	0.80 (0.44, 1.42)
				-	-	Continuous	-	0.94 (0.80, 1.09)
	Brender 2004	Case-control	-	-	-	< 3.52		1
			-	-	-	≥ 3.52		7.8 (1.6, 74.6)
	Brender 2013	Case-control	30	367	397	< 0.65	1	1
			42	360	402	0.65 to 3.5	1.43 (0.87, 2.33)	1.41 (0.86, 2.32)
			62	374	436	> 3.5	2.03 (1.28, 3.21)	2.02 (1.27, 3.22)
Anencephaly	Stayner 2022	Cohort	12	-	-	Continuous	-	1.19 (0.70, 2.01)

**Outcome**	**Study**	**Study design**	**Case**	**Controls**	**Total**	**Exposure groups NO_3-N (mg/L)**	**Unadjusted OR/MD (95%CI)**	**Adjusted OR/MD (95% CI)**
	Brender 2004	Case-control	-	-	-	< 0.80		1
			-	-	-	≥ 0.80		1.0 (0.3, 2.7)
	Brender 2013	Case-control	31	367	398	< 0.65	1	1
			17	360	377	0.65 to 3.5	0.56 (0.30, 1.03)	0.58 (0.32, 1.08)
			23	374	397	> 3.5	0.73 (0.42, 1.27)	0.78 (0.44, 1.37)
Encephalocele	Stayner 2022	Cohort	62	-	-	< 0.45	-	1
				-	-	0.45 to <1.13	-	0.77 (0.43, 1.37)
				-	-	1.13 to <5.65	-	0.49 (0.19, 1.24)
				-	-	≥ 5.65	-	-
				-	-	Continuous	-	0.92 (0.60, 1.41)
Microcephalus	Stayner 2022	Cohort	395	-	-	< 0.45	-	1
				-	-	0.45 to <1.13	-	1.00 (0.80, 1.26)
				-	-	1.13 to <5.65	-	0.87 (0.62, 1.20)
				-	-	≥ 5.65	-	0.69 (0.38, 1.26)
				-	-	Continuous	-	0.88 (0.75, 1.03)
Hydrocephalus	Stayner 2022	Cohort	501	-	-	< 0.45	-	1
				-	-	0.45 to <1.13	-	1.06 (0.87, 1.29)

Outcome	Study	Study design	Case	Controls	Total	Exposure groups NO_3-N (mg/L)	Unadjusted OR/MD (95%CI)	Adjusted OR/MD (95% CI)
				-	-	1.13 to <5.65	-	1.08 (0.83, 1.40)
				-	-	≥ 5.65	-	0.63 (0.35, 1.12)
				-	-	Continuous	-	0.92 (0.81, 1.04)
	Liu 2008	Case-control	-	-	-	Continuous	1.02 (0.94, 1.12)	Not significant
Any congenital heart defects	Cedergren 2002	Cohort study	316	26,097	26,413	< 0.45[a]	1	1
			409	29,002	29,411	≥ 0.45[a]	1.17 (1.0, 1.36)	1.18 (0.97, 1.44)
	Stayner 2022	Cohort study	9,752	-	-	< 0.45	1	1
				-	-	0.45 to <1.13	0.94 (0.90, 0.98)	0.94 (0.90, 0.99)
				-	-	1.13 to <5.65	0.98 (0.92, 1.04)	1.01 (0.95, 1.08)
				-	-	≥ 5.65	1.02 (0.92, 1.13)	1.04 (0.94, 1.15)
				-	-	Continuous	1.00 (0.98, 1.03)	1.01 (0.99, 1.04)
Conotruncal heart defects	Brender 2013	Case-control	58	370	428	< 0.71	1	1
			41	367	408	0.71 to 3.86	0.71 (0.47, 1.09)	0.72 (0.47, 1.11)
			65	368	433	> 3.86	1.13 (0.77, 1.65)	1.18 (0.80, 1.74)
Patent ductus arteriosus	Liu 2008	Case-control	-	-	-	Continuous	0.98 (0.88, 1.11)	-
Right ventricular outflow tract obstruction	Brender 2013	Case-control	36	370	406	< 0.71	1	1
			31	367	398	0.71 to 3.86	0.87 (0.53, 1.43)	0.89 (0.54, 1.48)

Outcome	Study	Study design	Case	Controls	Total	Exposure groups NO_3-N (mg/L)	Unadjusted OR/MD (95%CI)	Adjusted OR/MD (95% CI)
			53	368	421	> 3.86	1.48 (0.95, 2.32)	1.47 (0.93, 2.33)
Left ventricular outflow tract obstruction	Brender 2013	Case-control	44	370	414	< 0.71	1	1
			58	367	425	0.71 to 3.86	1.33 (0.88, 2.02)	1.31 (0.86, 2.00)
			54	368	422	> 3.86	1.23 (0.81, 1.88)	1.16 (0.75, 1.78)
Septal defects	Brender 2013	Case-control	203	370	573	< 0.71	1	1
			210	367	577	0.71 to 3.86	1.04 (0.82, 1.33)	0.92 (0.69, 1.22)
			156	368	524	> 3.86	0.76 (0.59, 0.98)	0.98 (0.71, 1.34)
Atrial septal defects	Liu 2008	Case-control	-	-	-	Continuous	0.97 (0.89, 1.05)	-
Ventricular septal defects	Liu 2008	Case-control	-	-	-	Continuous	0.85 (0.87, 1.04)	-
Tetralogy of fallot	Liu 2008	Case-control	-	-	-	Continuous	0.92 (0.76, 1.13)	-
Down syndrome	Liu 2008	Case-control	-	-	-	Continuous	0.88 (0.79, 1.0)	0.64 (0.49, 0.84)

Supplement Table S2. Characteristics of other type of studies.

Study name	Country, Region	Study design	Years of Outcome Ascertainment	Exposure description	Perinatal outcomes reported	Summary of findings
Blake 2014[1]	USA, California	Ecological	2011	Drinking water source data in the study ZIP codes were accessed from the California State Ground Water Ambient Monitoring and Assessment Program (GAMA) (California Water Board [CWB], 2013), a public geo-database that provides locations and water quality data by ZIP code and address.<br>Unsafe nitrate levels at ZIP code level: > 10 mg/L as NO_3-N	Low birth weight	ZIP codes with more dairy farms and a higher dairy cow density had higher levels of nitrate contamination. No correlation was detected between low birth weight and unsafe nitrate levels at the ZIP code level.
Blaisdell 2019[2]	USA, Missouri	Ecological	2004-2008	Average monthly concentrations of nitrate in drinking water were calculated from the finished water (water that has passed through all the processes in a water treatment plant) measurements taken from each Missouri community water system during the years 2004 – 2008. Monthly county-level average nitrate concentrations were linked to each birth by county and month of birth to estimate mean exposure during the 12 months prior to birth and during the first trimester of pregnancy.<br>Range of nitrate concentrations reported: 0.03 to 6.36 mg/L as NO_3-N	Neural tube defects, congenital heart defects, oral cleft defects, limb deficiencies, gastroschisis, hypospadias, Down Syndrome	Antenatal exposure to nitrate in drinking water was only associated with an increased rate of limb deficiencies. Rate Ratio for 1 mg/L = 1.26, 95% CI 1.05, 1.51
Bukowski 2001[3]	Canada, Prince Edward Island	Ecological	1991-1994	Individual postal codes were combined into 47 polygons based on contiguous postal codes that used similar water sources. The polygons were combined into six exposure groups of increasing nitrate	Intrauterine growth registration, preterm birth	The higher nitrate exposure categories (median nitrate concentration >3.1 mg/L) were positively associated with low birth weight odds ratio: 2.40 (95% CI 1.75,

				concentrations. Maternal residential postal code at the time of delivery matched to nitrate exposure groups.<br>Exposure measured period: not specified, samples assumed to represent exposure during pregnancy.<br>Range of nitrate concentrations reported: 0 to 37.5 mg/L as NO_3-N.		3.27) and preterm birth odds ratio: 1.91 (95% CI 1.47, 2.46).
Huang 2018[4]	USA, California	Cross-sectional study	2009-2012	Birth cohort file from the California Office of Statewide Health linked to CalEnviro Screen 3.0 dataset from California Communities Environmental Health Screening Tool.<br>Range of nitrate level reported: 1.41 - 85.48 mg/L as NO_3^- or 0.32 - 19.30 mg/L as NO_3-N	Preterm birth	Nitrate in drinking water was potentially associated with preterm birth in California.<br>Odds ratio per increase in interquartile range (9.33 mg/L NO_3^- or 2.11 mg/L NO_3-N) was 1.02 (95 % CI 1.01, 1.03)
Joyce 2008[5]	Australia, Perth	Record-based prevalence study	2002-2004	Maternal residential addresses at the time of birth were assigned to a collection district and water distribution zone code for water contaminant measurements.<br>Nitrate concentrations were classified as low (< 0.125 mg/L), median (0.125-0.350 mg/L) or high (> 3.50 mg/L)	Prelabour rupture of membranes	Increasing exposure to nitrate in drinking water was significantly associated with an increased risk of prelabour rupture of membranes.<br>Adjusted odds ratios for prelabour rupture of membranes increased with increasing tertiles of nitrate exposure (moderate exposure: odds ratio: 1.23 (95 % CI 1.03, 1.52); high exposure: odds ratio 1.47 (95% CI 1.20, 1.79).
Mattix 2007[6]	USA, Indiana	Ecological study	1990- 2002	Monthly abdominal wall defect rates linked to monthly surface water nitrate concentration<br>Nitrate concentrations reported: below 5 mg/L as NO_3-N.	Abdominal wall birth defects	No correlation observed between nitrate levels in surface water and monthly abdominal wall defects rate.

Ouattara 2022[7]	USA, Nebraska	Ecological study	1995-2014	Nebraska has 23 natural resource districts. Groundwater samples from private wells were collected from various district areas. Samples were collected between June 2021 and Feb 2022.<br><br>Range of nitrate level reported: < 6.94 mg/L or >6.94 mg/L as NO_3-N	Any birth defect	A positive association was observed between high levels of nitrate in drinking water and the prevalence of birth defects.
Padula 2021[8]	USA, California	Cross-sectional study	2007- 2012	California birth certificates and maternal and infant hospital discharge records from the Office of Statewide Health Planning and Development linked to CalEnviro Screen 3.0 dataset from California Communities Environmental Health Screening Tool.<br><br>Range of nitrate level reported: 1.41 - 85.48 mg/L as NO_3^- mg/L or 0.32 - 19.30 mg/L as NO_3-N	Gestational hypertension, eclampsia	Nitrate was not associated with hypertensive disorders, severe preeclampsia/ eclampsia in pregnancy in the single pollutant model.<br><br>Nitrate was associated with hypertensive disorders, severe preeclampsia/ eclampsia in pregnancy only in the multipollutant model including all contaminates (arsenic, cadmium,1,2-Dibromo-3-chloropropane, hexavalent chromium, lead, nitrate, perchlorate, tetachloroethylene, radium, trichloroethylene, 1,2,3-trichloropropane, trihalomethane, uranium)
Stayner 2017[9]	USA,<br><br>Indiana,<br><br>Iowa, Missouri, and Ohio	Ecological	2004–2008	Average monthly county-level mean nitrate concentrations were calculated from finished water (water that has passed through all the processes in a water treatment plant) for each community water system and weighted by the population; Monthly county-level average nitrate concentrations were linked to each birth.	Preterm birth<br><br>Low birth weight	There were no associations between antenatal exposure to nitrate in drinking water and preterm birth or low birth weight overall.

				The mean of the monthly county-level nitrate concentrations was 0.95 ± 0.92 mg/L as NO_3-N. 1.8% of the monthly estimates exceeded 10 mg/L as NO_3-N		
Super 1981[10]	Southwest Africa/Namibia	Cross-sectional study	Unclear	Water from the wells that parents used were taken for analysis, which included nitrate levels.<br><br>Regions were classified as low nitrate (≤ 4.518 mg/L as NO_3-N) or high nitrate (> 4.518 mg/L as NO_3-N)	Preterm birth<br>Size of infant at birth (reported by mother)	There were no associations between high nitrate regions and preterm birth or size of infant at birth.
Winchester 2009[11]	USA, national-wide	Ecological	1996-2002	Monthly pregnancy and birth outcome data from the Centers for Disease Control (CDC) natality database linked to the monthly USGS (The United States Geological Survey) and NAWQA (National Water Quality Assessment Programme) databases.<br>The mean nitrate concentration in April to July was 1.31 ± 0.20 mg/L as NO_3-N, and in other months was 0.16 ± 0.02 as NO_3-N.	Birth defects including 22 birth defect categories	There were no associations between the months of increased levels of nitrates (April-July) and spina bifida, oral cleft, circulation system defects, Down syndrome, gastroschisis, urogenital defects and clubfoot or oral cleft.

Supplement Note S1. PRISMA Checklist

Section and Topic	Item #	Checklist item	Location where item is reported
**TITLE**			
Title	1	Identify the report as a systematic review.	Title
**ABSTRACT**			
Abstract	2	See the PRISMA 2020 for Abstracts checklist.	Abstract
**INTRODUCTION**			
Rationale	3	Describe the rationale for the review in the context of existing knowledge.	Introduction P1-P4
Objectives	4	Provide an explicit statement of the objective(s) or question(s) the review addresses.	Introduction P5
**METHODS**			
Eligibility criteria	5	Specify the inclusion and exclusion criteria for the review and how studies were grouped for the syntheses.	Methods: Criteria for considering studies for this review
Information sources	6	Specify all databases, registers, websites, organisations, reference lists and other sources searched or consulted to identify studies. Specify the date when each source was last searched or consulted.	Methods: Search strategy
Search strategy	7	Present the full search strategies for all databases, registers and websites, including any filters and limits used.	Supplement 3
Selection process	8	Specify the methods used to decide whether a study met the inclusion criteria of the review, including how many reviewers screened each record and each report retrieved, whether they worked independently, and if applicable, details of automation tools used in the process.	Methods: study selection
Data collection process	9	Specify the methods used to collect data from reports, including how many reviewers collected data from each report, whether they worked independently, any processes for obtaining or confirming data from study investigators, and if applicable, details of automation tools used in the process.	Methods: data extraction
Data items	10a	List and define all outcomes for which data were sought. Specify whether all results that were compatible with each outcome domain in each study were sought (e.g. for all measures, time points, analyses), and if not, the methods used to decide which results to collect.	Methods: data extraction & Supplement 4
	10b	List and define all other variables for which data were sought (e.g. participant and intervention characteristics, funding sources). Describe any assumptions made about any missing or unclear information.	Methods: data extraction & Supplement 4
Study risk of bias assessment	11	Specify the methods used to assess risk of bias in the included studies, including details of the tool(s) used, how many reviewers assessed each study and whether they worked independently, and if applicable, details of automation tools used in the process.	Methods: study quality
Effect measures	12	Specify for each outcome the effect measure(s) (e.g. risk ratio, mean difference) used in the synthesis or	Methods: statistical analysis

Section and Topic	Item #	Checklist item	Location where item is reported
		presentation of results.	
Synthesis methods	13a	Describe the processes used to decide which studies were eligible for each synthesis (e.g. tabulating the study intervention characteristics and comparing against the planned groups for each synthesis (item #5)).	Methods: study quality
	13b	Describe any methods required to prepare the data for presentation or synthesis, such as handling of missing summary statistics, or data conversions.	Methods: statistical analysis
	13c	Describe any methods used to tabulate or visually display results of individual studies and syntheses.	Methods: statistical analysis
	13d	Describe any methods used to synthesize results and provide a rationale for the choice(s). If meta-analysis was performed, describe the model(s), method(s) to identify the presence and extent of statistical heterogeneity, and software package(s) used.	Methods: statistical analysis
	13e	Describe any methods used to explore possible causes of heterogeneity among study results (e.g. subgroup analysis, meta-regression).	Methods: statistical analysis
	13f	Describe any sensitivity analyses conducted to assess robustness of the synthesized results.	Methods: statistical analysis
Reporting bias assessment	14	Describe any methods used to assess risk of bias due to missing results in a synthesis (arising from reporting biases).	Methods: statistical analysis
Certainty assessment	15	Describe any methods used to assess certainty (or confidence) in the body of evidence for an outcome.	N/A
**RESULTS**			
Study selection	16a	Describe the results of the search and selection process, from the number of records identified in the search to the number of studies included in the review, ideally using a flow diagram.	Results:<br>Search results and study characteristics
	16b	Cite studies that might appear to meet the inclusion criteria, but which were excluded, and explain why they were excluded.	Results:<br>Search results and study characteristics; Figure 1
Study characteristics	17	Cite each included study and present its characteristics.	Table 1
Risk of bias in studies	18	Present assessments of risk of bias for each included study.	Table 2
Results of individual studies	19	For all outcomes, present, for each study: (a) summary statistics for each group (where appropriate) and (b) an effect estimate and its precision (e.g. confidence/credible interval), ideally using structured tables or plots.	table 4
Results of	20a	For each synthesis, briefly summarise the characteristics and risk of bias among contributing studies.	Discussion P3

Section and Topic	Item #	Checklist item	Location where item is reported
syntheses	20b	Present results of all statistical syntheses conducted. If meta-analysis was done, present for each the summary estimate and its precision (e.g. confidence/credible interval) and measures of statistical heterogeneity. If comparing groups, describe the direction of the effect.	Results, table 4
	20c	Present results of all investigations of possible causes of heterogeneity among study results.	Discussion P3
	20d	Present results of all sensitivity analyses conducted to assess the robustness of the synthesized results.	N/A
Reporting biases	21	Present assessments of risk of bias due to missing results (arising from reporting biases) for each synthesis assessed.	N/A
Certainty of evidence	22	Present assessments of certainty (or confidence) in the body of evidence for each outcome assessed.	N/A
**DISCUSSION**			
Discussion	23a	Provide a general interpretation of the results in the context of other evidence.	Discussion: p1,p2,p5, p6
	23b	Discuss any limitations of the evidence included in the review.	Discussion: p11
	23c	Discuss any limitations of the review processes used.	Discussion: p11
	23d	Discuss implications of the results for practice, policy, and future research.	Discussion: p7-10
**OTHER INFORMATION**			
Registration and protocol	24a	Provide registration information for the review, including register name and registration number, or state that the review was not registered.	Methodology: p1
	24b	Indicate where the review protocol can be accessed, or state that a protocol was not prepared.	Methodology: p1
	24c	Describe and explain any amendments to information provided at registration or in the protocol.	N/A
Support	25	Describe sources of financial or non-financial support for the review, and the role of the funders or sponsors in the review.	Acknowledgements
Competing interests	26	Declare any competing interests of review authors.	Title page
Availability of data, code and other materials	27	Report which of the following are publicly available and where they can be found: template data collection forms; data extracted from included studies; data used for all analyses; analytic code; any other materials used in the review.	Methodology and supplement

From: Page MJ, McKenzie JE, Bossuyt PM, Boutron I, Hoffmann TC, Mulrow CD, et al. The PRISMA 2020 statement: an updated guideline for reporting systematic reviews. BMJ 2021;372:n71. doi: 10.1136/bmj.n71

For more information, visit: http://www.prisma-statement.org/

Supplement Note S2. Protocol

Nitrate contamination in drinking water and adverse reproductive outcomes: protocol for a systematic review and meta-analysis

Background

Nitrate is a naturally occurring ion that is part of the nitrogen cycle and used mainly in inorganic fertilisers. The amount of nitrate ingested from drinking water varies based on its concentration in drinking water and an individual's consumption habits. The increasing use of artificial fertilisers, the disposal of wastes, particularly from animal farming, and changes in land use have become significant contributors to the progressive increase in nitrate levels in groundwater supplies[12].

While there is some evidence that nitrates in drinking water are associated with colorectal cancer[13, 14], the potential adverse reproductive effects of chronic exposure to low levels of nitrate have been emphasised recently. Animal studies have indicated that nitrate from the mother can cross the placenta, affect the foetus in utero, and increases adverse outcomes [15-17]. In addition, several epidemiological studies have indicated associations between prenatal nitrate exposure and other adverse reproductive outcomes, including congenital abnormalities, preterm birth, low birth weight and small-for-gestational-age (SGA)[18-22].

Therefore, the purpose of this study is to systematically review the available data and assess the association between human exposure to nitrate in drinking water and adverse reproductive outcomes.

Methods

Criteria for considering studies for this review

We will include randomised trials, cohort and case-control studies published in English. Studies that report the relationship between nitrate intake from drinking water and the risk of perinatal outcomes will be included.

The exposure of interest is nitrate intake from drinking water during the antenatal period.

The primary outcome for this review will be a composite of any of the following outcomes: preterm birth; SGA infant; low birth weight infant; stillbirth; miscarriage; and neonatal death.

The secondary outcomes will be:

For infants: preterm birth, SGA, low birth weight, stillbirth, neonatal death, perinatal death, hypoglycaemia, need for respiratory support after birth, infection, congenital abnormality, necrotising enterocolitis, bronchopulmonary dysplasia, intraventricular haemorrhage, neonatal lung disease, neonatal intensive care unit (NICU) admission, jaundice, methemoglobinemia (as defined by the authors).

For women: any pregnancy complications (miscarriage, high blood pressure, preeclampsia, gestational diabetes, infection, obstetric haemorrhage as defined by the authors).

Search strategy

We will conduct a comprehensive search of databases from inception to current, including Ovid MEDLINE via PubMed, Embase, CINAHL, Cochrane Central Register of Controlled Trials (CENTRAL, current issue) in the Cochrane Library, Web of Science, Scopus, GEOBASE and ProQuest Agricultural and Environmental Science Database, using search terms unique to the review topic. We will search using both English and American spelling. We will not apply language restrictions, but only full text in English will be included.

Additionally, we will review the reference lists of all identified articles for relevant articles not identified in the primary search.

Selection of studies

Two authors will independently evaluate and appraise the retrieved studies using COVIDENCE [23], following the steps below.

1. Import all the records from the database into COVIDENCE;
2. Screen titles and abstracts to select relevant reports and exclude studies not relevant for this review;
3. Examine full-text studies for compliance with the eligibility criteria determined for this review;
4. Make final decisions on study inclusion and proceed to data collection.

We will resolve disagreements by discussion and if necessary, a third review author will mediate for differences in interpretation.

We will record the selection process in sufficient detail to complete a PRISMA flow diagram[24].

Data extraction and management

We will develop a data extraction form to extract data from eligible studies. Two review authors will independently extract data from each eligible study. Information extracted will include, but not be limited to: source details, eligibility assessment, methodological details, characteristics of participants, details of intervention and outcomes reported. Any disagreement will be resolved by discussion and if necessary in discussion with a third review author.

Assessment of risk of bias in included studies

We will assess the quality of case-control and cohort studies according to the Newcastle-Ottawa Scale (NOS) [25]. The NOS evaluates nine methodological items and their reporting (participant selection, comparability of groups, and ascertainment of exposure/outcome), with values ≥ 7 compatible with good study quality (least bias, results are considered valid), between 2 and 7 with moderate study quality (susceptible to some bias but probably not enough to invalidate the results), and ≤ 2 with poor study quality (significant bias that may invalidate results).

We will assess the quality of randomized trials using the methods specified in the Cochrane Handbook for Systematic Reviews of Interventions[26]: (1) random sequence generation (selection bias); (2) allocation concealment (selection bias); (3) blinding of participants, personnel and outcome assessment (performance and detection bias); (4) incomplete outcome data (attrition bias); (5) selective reporting (reporting bias); (6) other bias (checking for bias due to problems not covered by (1) to (5) above).

Statistical analysis

The relationship between nitrate intake from drinking water and the risk of adverse birth outcomes will be examined based on the effect estimate. Nitrate intake from drinking water, odds ratios (ORs), risk ratios (RRs), hazard ratios (HRs), with 95% confidence intervals (CIs) will be extracted (both crude and adjusted). The median intake of nitrate will be calculated from the range given, (median intake = lowest dosage (lower limit) + highest dosage (upper limit) divided by 2 in each given quartile). When the median intake is given, the data will be used directly. When the interval of a quartile of any category of nitrate intake is not provided, the width of the class interval of quartile before this quartile will be used to calculate and estimate the interval, (when the lowest or highest category is open-ended, we assume that the open-ended interval length has the same length as the adjacent interval).

Meta-regression analysis will be conducted to assess the associations between nitrate exposure and adverse outcomes and $p < 0.05$ will denote statistical significance for a positive meta-regression coefficient. The pooled results across included studies will be evaluated using a random-effects model. Heterogeneity test will be performed using the I-square and Q-statistic, and significant heterogeneity is defined as $p < 0.010$.

Statistical analyses will be performed using STATA.

Discussion

This systematic review and meta-analysis, using the available evidence, will assess whether there is an association between nitrate in drinking water and the risk of adverse birth outcomes. The findings may help guide policy decisions regarding the safe level of nitrate in drinking water.

Supplement Note S3. Search strategies

a. Search strategies for Ovid MEDLINE, CINAHL Plus and Cochrane Central

1 Nitrates/

2 nitrate*.af.

3 1 or 2

4 Drinking Water/

5 water supply/ or water wells/

6 Water/

7 groundwater/

8 (groundwater or aquifer*).ti,ab,kw.

9 water.ti,ab,kw,kf.

10 or/4-9

11 exp Pregnancy/

12 exp Pregnancy Complications/

13 Maternal-Fetal Exchange/ 29788

14 (maternal fetal exchange or maternal foetal exchange).mp.

15 ((transplacent* or trans-placent*) adj (exposure or exchange)).mp.

16 (stillbirth* or still-birth*).ti,ab,kw.

17 (preeclamp* or pre-eclamp* or eclamp*).ti,ab,kw.

18 ((pregnan* or postpartum* or post-partum* or perinatal* or peri-natal* or puerperal) and (complication* or infecti* or haemorrhag* or hemorrhag* or sepsis or septic)).ti,ab,kw,kf.

19 ((neonatal or neo-natal or perinatal or peri-natal) adj death*).ti,ab,kw.

20 or/11-19

21 infant, small for gestational age/ or infant, very low birth weight/ or infant, extremely low birth weight/ or infant, premature/ or infant, extremely premature/

22 (low adj (birth weight* or birthweight*)).mp.

23 (sga or "small for gestational age").mp.

24 ((fetal or foetal or intrauterin* or intra-uterin*) adj3 (restrict* or retard*)).mp.

25 ((preterm or pre-term or prematur*) adj2 (birth or births or born or labour or labor)).ti,ab,kw.

26 (prematurity or congenital or spontaneous abort*).mp.

27 ((preterm or pre-term or prematur*) adj2 (baby or babies or infant or infants)).ti,ab,kw.

28 infant, premature, diseases/ or bronchopulmonary dysplasia/ or leukomalacia, periventricular/ or respiratory distress syndrome, newborn/ or hyaline membrane disease/ or "transient tachypnea of the newborn"/

29 jaundice, neonatal/ or neonatal sepsis/

30 ((intraventricular or intra-ventricular) adj3 (hemorrhag* or haemorrhag*)).mp.

31 (severe brain injur* and (infant* or baby or babies or prematur* or newborn* or new* born* or neonat* or neo-nat* or preterm or pre-term)).mp.

32 Enterocolitis, Necrotizing/

33 Hypoglycemia/

34 (bronchopulmonary dysplasia* or broncho-pulmonary dysplasia*).ti,ab,kw.

35 (necroti* enterocolitis or necroti* entero-colitis).ti,ab,kw.

36 ((hypoglyc* or respirat* or infection* or sepsis or septic or jaundice or lung or lungs) and (infant* or baby or babies or prematur* or newborn* or new* born* or neonat* or neo-nat* or preterm or pre-term)).mp.

37 Intensive Care, Neonatal/

38 (neonatal intensive care or nicu).ti,ab,kw.

39 or/21-38

40 20 or 39

41 3 and 10 and 40

b. Search strategies for Embase

1 nitrate/

2 nitrate*.af.

3 1 or 2

4 drinking water/

5 water supply/ or water wells/

6 water/ or drinking water/ or ground water/ or tap water/ or well water/

7 ((drinking or potable or bottled or well) adj water).mp.

8 (water adj (supply or supplies or well or wells)).mp.

9 or/4-8

10 exp Pregnancy/

11 exp Pregnancy Complications/

12 Maternal-Fetal Exchange/

13 (maternal adj (fetal or foetal) adj exchange).mp.

14 ((transplacent* or trans-placent*) adj (exposure or exchange)).mp.

15 (stillbirth* or still-birth*).ti,ab,kw.

16 (preeclamp* or pre-eclamp* or eclamp*).ti,ab,kw.

17 ((pregnan* or postpartum* or post-partum* or perinatal* or peri-natal* or puerperal) and (complication* or infecti* or haemorrhag* or hemorrhag* or sepsis or septic)).ti,ab,kw.

18 ((neonatal or neo-natal or perinatal or peri-natal) adj death*).ti,ab,kw.

19 or/10-18

20 infant, small for gestational age/ or infant, very low birth weight/ or infant, extremely low birth weight/ or infant, premature/ or infant, extremely premature/

21 (low adj (birth weight* or birthweight*)).mp.

22 (sga or "small for gestational age").mp.

23 ((fetal or foetal or intrauterin* or intra-uterin*) adj3 (restrict* or retard*)).mp.

24 ((preterm or pre-term or prematur*) adj2 (birth or births or born or labour or labor)).ti,ab,kw.

25 prematurity.mp.

26 ((preterm or pre-term or prematur*) adj2 (baby or babies or infant or infants)).ti,ab,kw.

27 infant, premature, diseases/ or bronchopulmonary dysplasia/ or leukomalacia, periventricular/ or respiratory distress syndrome, newborn/ or hyaline membrane disease/ or "transient tachypnea of the newborn"/

28 jaundice, neonatal/ or neonatal sepsis/

29 ((intraventricular or intra-ventricular) adj3 (hemorrhag* or haemorrhag*)).mp.

30 (severe brain injur* and (infant* or baby or babies or prematur* or newborn* or new* born* or preterm or pre-term or neonat* or neo-nat*)).mp.

31 necrotizing enterocolitis/

32 hypoglycemia/

33 (bronchopulmonary dysplasia* or broncho-pulmonary dysplasia*).ti,ab,kw.

34 (necroti* enterocolitis or necroti* entero-colitis).ti,ab,kw.

35 ((hypoglyc* or respirat* or infection* or sepsis or septic or jaundice) and (infant* or baby or babies or prematur* or newborn* or new* born* or preterm or pre-term or neonat* or neo-nat*)).mp.

36 or/20-35

37 19 or 36

38 3 and 9 and 37

c. Search strategies for GEOBASE and Agricultural & Environmental Science Database via ProQuest

noft(nitrate*) AND noft(groundwater OR aquifer* OR water) AND ((noft(maternal fetal exchange OR maternal foetal exchange) OR noft((transplacent* OR trans-placent*) NEAR/1 (exposure OR exchange)) OR noft(stillbirth* OR still-birth*) OR noft(preeclamp* OR pre-eclamp* OR eclamp*) OR noft((neonatal OR neo-natal OR perinatal OR peri-natal) NEAR/1 death*) OR noft(congenital) OR noft("spontaneous abort*") OR (noft((pregnan* OR postpartum* OR post-partum* OR perinatal* OR peri-natal* OR puerperal) AND (complication* OR infecti* OR haemorrhag* OR hemorrhag* OR

sepsis OR septic)) OR noft(low NEAR/1 ("birth weight*" OR birthweight*)) OR noft(sga OR "small for gestational age") OR noft((fetal OR foetal OR intrauterin* OR intra-uterin*) NEAR/3 (restrict* OR retard*)) OR noft((preterm OR pre-term OR prematur*) NEAR/2 (birth OR births OR born OR labour OR labor)) OR noft(prematurity) OR noft((preterm OR pre-term OR prematur*) NEAR/2 (baby OR babies OR infant OR infants))) OR (noft((intraventricular OR intra-ventricular) NEAR/3 (hemorrhag* OR haemorrhag*)) OR noft("severe brain injur*" AND (infant* OR baby OR babies OR prematur* OR newborn* OR "new*born*" OR neonat* OR neo-nat* OR preterm OR pre-term)) OR noft("bronchopulmonary dysplasia*" OR "broncho-pulmonary dysplasia*") OR noft("necroti* enterocolitis" OR "necroti* entero-colitis") OR noft((hypoglyc* OR respirat* OR infection* OR sepsis OR septic OR jaundice OR lung OR lungs) AND (infant* OR baby OR babies OR prematur* OR newborn* OR "new* born*" OR neonat* OR neo-nat* OR preterm OR pre-term)) OR noft("neonatal intensive care" OR nicu)))

CINAHL Plus

(nitrate*) AND (groundwater OR aquifer* OR water) AND ((maternal fetal exchange OR maternal foetal exchange) OR ((transplacent* OR trans-placent*) NEAR/1 (exposure OR exchange)) OR (stillbirth* OR still-birth*) OR (preeclamp* OR pre-eclamp* OR eclamp*) OR ((neonatal OR neo-natal OR perinatal OR peri-natal) NEAR/1 death*) OR (congenital) OR ("spontaneous abort*") OR ((pregnan* OR postpartum* OR post-partum* OR perinatal* OR peri-natal* OR puerperal) AND (complication* OR infecti* OR haemorrhag* OR hemorrhag* OR sepsis OR septic)) OR (low NEAR/1 ("birth weight*" OR birthweight*)) OR (sga OR "small for gestational age") OR ((fetal OR foetal OR intrauterin* OR intra-uterin*) NEAR/3 (restrict* OR retard*)) OR ((preterm OR pre-term OR prematur*) NEAR/2 (birth OR births OR born OR labour OR labor)) OR noft(prematurity) OR ((preterm OR pre-term OR prematur*) NEAR/2 (baby OR babies OR infant OR infants))) OR (noft((intraventricular OR intra-ventricular) NEAR/3 (hemorrhag* OR haemorrhag*)) OR ("severe brain injur*" AND (infant* OR baby OR babies OR prematur* OR newborn* OR "new*born*" OR neonat* OR neo-nat* OR preterm OR pre-term)) OR ("bronchopulmonary dysplasia*" OR "broncho-pulmonary dysplasia*") OR ("necroti* enterocolitis" OR "necroti* entero-colitis") OR ((hypoglyc* OR respirat* OR infection* OR sepsis OR septic OR jaundice OR lung OR lungs) AND (infant* OR baby OR babies OR prematur* OR newborn* OR "new* born*" OR neonat* OR neo-nat* OR preterm OR pre-term)) OR ("neonatal intensive care" OR nicu)))

Supplement Note S4. Data Extraction Form

Data Extraction Form

Review title: Nitrate contamination in drinking water and adverse birth outcomes

**Study ID**:		
**Person extracting data:**	**Date of date extraction:**	**Year of study publication:**
**Title:**		
**Author:**		
**Reference:**		
**Other publications from same study (**additional reports of the same study should be grouped under the same study identifier see "Organising studies and references", p 35 RevMan User Guide**):**		

Study eligibility

Study Characteristics	Eligibility criteria *(Insert inclusion criteria for each characteristic as defined in the Protocol)*	Eligibility criteria met? (add X to select decision) Yes	No	Unclear	Location in text or source *(pg & ¶/fig/table/other)*
Type of study	Case-control or Cohort study				

Participants	Pregnant women- neonatal				
Types of intervention (exposure)	Nitrate in drinking water				
Types of comparison					
Types of outcome measures	Perinatal outcome				
DECISION: Potentially include? YES/NO Exclude YES/NO					
Reason for exclusion					
Notes:					

DO NOT PROCEED IF STUDY EXCLUDED FROM REVIEW

Study design and general characteristics

Type of study design:<br><br>Location:<br><br>Study dates:<br><br>Unit of allocation (by individuals, cluster/groups or body parts):<br><br>Study funding sources:<br><br>Study authors' declarations of interest:<br><br>Ethics approval

Population and setting

Describe setting (including location and social context)

Water sample description:

Population description:

Inclusion criteria:

Exclusion criteria:

Notes:

Intervention

Exposure:

Duration of exposure:

Total number of participants: n=

Comparison

Control/Comparison:

Total number of participants: n=

Outcomes:

Outcomes:

Outcomes for main analysis

**Dichotomous outcome**	**Description as stated in report/paper**			
**Outcome**				
**Results**	**Intervention**		**Comparison**	
	No. events	No. participants	No. events	No. participants
**Risk ratio or odds ratio**<br>*Note whether*<br>**...** *Adjusted OR*<br>**...***Unadjusted*				
**Unit of analysis**<br>*(e.g. by individuals, health professional, practice, hospital, community)*				
**Statistical methods used and appropriateness of these methods**<br>*(e.g. adjustment for correlation)*				
**Notes:**				

<table>
<tr><td>Continues outcome</td><td colspan="6">Description as stated in report/paper</td></tr>
<tr><td>Outcome</td><td colspan="6"></td></tr>
<tr><td rowspan="3">Results
Not ewhether
... Adjusted OR
...Unadjusted</td><td colspan="3">Intervention</td><td colspan="3">Comparison</td></tr>
<tr><td>Mean</td><td>SD (or other variance)</td><td>No. participants</td><td>Mean</td><td>SD (or other variance)</td><td>No. participants</td></tr>
<tr><td></td><td></td><td></td><td></td><td></td><td></td></tr>
<tr><td>Unit of analysis
(e.g. by individuals, health professional, practice, hospital, community)</td><td colspan="6"></td></tr>
<tr><td>Statistical methods used and appropriateness of these methods
(e.g. adjustment for correlation)</td><td colspan="6"></td></tr>
<tr><td colspan="7">Notes:</td></tr>
</table>

General conclusions

**Very brief summary of the <u>study authors'</u> main findings/conclusions:**

Notes

Exclusion after data extraction

Reasons for exclusion: (study design? participants? interventions/ outcomes? attrition? bias?)

References

1. Blake SB. Spatial relationships among dairy farms, drinking water quality, and maternal-Child health outcomes in the San Joaquin Valley. Public Health Nursing. 2014;31(6):492-9.
2. Blaisdell J, Turyk ME, Almberg KS, Jones RM, Stayner LT. Prenatal exposure to nitrate in drinking water and the risk of congenital anomalies. Environmental Research. 2019;176:108553.
3. Bukowski J, Somers G, Bryanton J. Agricultural contamination of groundwater as a possible risk factor for growth restriction or prematurity. Journal of Occupational and Environmental Medicine. 2001;43(4):377-83.
4. Huang H, Woodruff TJ, Baer RJ, Bangia K, August LM, Jellife-Palowski LL, et al. Investigation of association between environmental and socioeconomic factors and preterm birth in California. Environment International. 2018;121:1066-78.
5. Joyce SJ, Cook A, Newnham J, Brenters M, Ferguson C, Weinstein P. Water disinfection by-products and prelabor rupture of membranes. American Journal of Epidemiology. 2008;168(5):514-21.
6. Mattix KD, Winchester PD, Scherer LRT. Incidence of abdominal wall defects is related to surface water atrazine and nitrate levels. Journal of Pediatric Surgery. 2007;42(6):947-9.
7. Ouattara BS, Zahid M, Rahman FI, Weber KA, Bartelt-Hunt SL, Rogan EG. Investigation of a Possible Relationship between Anthropogenic and Geogenic Water Contaminants and Birth Defects Occurrence in Rural Nebraska. Water [Internet]. 2022;14(15).
8. Padula AM, Ma C, Huang H, Morello-Frosch R, Woodruff TJ, Carmichael SL. Drinking water contaminants in California and hypertensive disorders in pregnancy. Environmental Epidemiology. 2021;5(2):e149.
9. Stayner LT, Almberg K, Jones R, Graber J, Pedersen M, Turyk M. Atrazine and nitrate in drinking water and the risk of preterm delivery and low birth weight in four Midwestern states. Environmental Research. 2017;152:294-303.
10. Super M, Heese HD, Mackenzie D, Dempster WS, Duplessis J, Ferreira JJ. An epidemiological-study of well-water nitrates in a group of south-west-African Namibian infants. Water Research. 1981;15(11):1265-70.
11. Winchester PD, Huskins J, Ying J. Agrichemicals in surface water and birth defects in the United States. Acta Paediatrica. 2009;98(4):664-9.
12. World Health Organization. Nitrate and nitrite in drinking-water: background document for development of WHO guidelines for drinking-water quality. Geneva: World Health Organization; 2003. Contract No.: WHO/SDE/WSH/04.03/56.
13. Schullehner J, Hansen B, Thygesen M, Pedersen CB, Sigsgaard T. Nitrate in drinking water and colorectal cancer risk: A nationwide population-based cohort study. International Journal of Cancer. 2018;143(1):73-9.
14. Ward MH, Jones RR, Brender JD, de Kok TM, Weyer PJ, Nolan BT, et al. Drinking water nitrate and human health: an updated review. International Journal of Environmental Research and Public Health. 2018;15(7):1557.
15. Manassaram DM, Backer LC, Moll DM. A review of nitrates in drinking water: Maternal exposure and adverse reproductive and developmental outcomes. Environ Health Perspect. 2006;114(3):320-7.
16. Clausen HS, Ebdrup NH, Barsoe IM, Lyngso J, Schullehner J, Ramlau-Hansen CH, et al. Association between drinking water nitrate and adverse reproductive outcomes: a systematic PRISMA review. Water. 2020;12(8):2287.
17. Joshi N, Rhoades MG, Bennett GD, Wells SM, Mirvish SS, Breitbach MJ, et al. Developmental Abnormalities in Chicken Embryos Exposed to N-Nitrosoatrazine. Journal of Toxicology and Environmental Health-Part a-Current Issues. 2013;76(17):1015-22.
18. Almberg KS, Turyk M, Jones RM, Anderson R, Graber J, Banda E, et al. A study of adverse birth outcomes and agricultural land use practices in Missouri. Environmental Research. 2014;134:420-6.

19. Blaisdell J, Turyk ME, Almberg KS, Jones RM, Stayner LT. Prenatal exposure to nitrate in drinking water and the risk of congenital anomalies. Environmental Research. 2019;176.
20. Brender JD, Weyer PJ, Romitti PA, Mohanty BP, Shinde MU, Vuong AM, et al. Prenatal Nitrate Intake from Drinking Water and Selected Birth Defects in Offspring of Participants in the National Birth Defects Prevention Study. Environmental Health Perspectives. 2013;121(9):1083-9.
21. Coffman VR, Jensen AS, Trabjerg BB, Pedersen CB, Hansen B, Sigsgaard T, et al. Prenatal Exposure to Nitrate from Drinking Water and Markers of Fetal Growth Restriction: A Population-Based Study of Nearly One Million Danish-Born Children. Environmental Health Perspectives. 2021;129(2).
22. Holtby CE, Guernsey JR, Allen AC, VanLeeuwen JA, Allen VM, Gordon RJ. A population-based case-control study of drinking-water nitrate and congenital anomalies using geographic information systems (GIS) to develop individual-level exposure estimates. International Journal of Environmental Research and Public Health. 2014;11(2):1803-23.
23. Covidence systematic review software. Melbourne, Australia: Veritas Health Innovation. Available from: www.covidence.org
24. Moher D, Liberati A, Tetzlaff J, Altman DG, Group P. Preferred reporting items for systematic reviews and meta-analyses: the PRISMA statement. PLoS Medicine. 2009;6(7):e1000097.
25. Wells G, Shea B, O'Connell D, Peterson J, Welch V, Losos M, et al. The Newcastle-Ottawa Scale (NOS) for assessing the quality of nonrandomised studies in meta-analyses. Ottawa Hospital Research Institute 2013 [August 25, 2021]. Available from: http://www.ohri.ca/programs/clinical_epidemiology/oxford.asp
26. Higgins JPT, Thomas J, Chandler J, Cumpston M, Li T, Page MJ, et al. Cochrane Handbook for Systematic Reviews of Interventions version 6.3 (updated February 2022). 2022. Available from: www.training.cochrane.org/handbook.
